# Genome sequencing of *Prototheca zopfii* genotypes 1 and 2 provides evidence of a severe reduction in organellar genomes

**DOI:** 10.1038/s41598-018-32992-0

**Published:** 2018-10-02

**Authors:** Marco Severgnini, Barbara Lazzari, Emanuele Capra, Stefania Chessa, Mario Luini, Roberta Bordoni, Bianca Castiglioni, Matteo Ricchi, Paola Cremonesi

**Affiliations:** 1 Institute of Biomedical Technologies, National Research Council (ITB-CNR), Segrate, Milan, Italy; 2 PTP-Science Park, Lodi, Italy; 3 Institute of Agricultural Biology and Biotechnology, National Research Council (IBBA-CNR), Lodi, Italy; 4 Lombardy and Emilia Romagna Experimental Zootechnic Institute (IZSLER), Lodi, Italy; 5 Lombardy and Emilia Romagna Experimental Zootechnic Institute (IZSLER), Piacenza, Italy

**Keywords:** Zopfii, Helicosporidium, Plastid Genome, Trebouxiophyceae, Nuclear-encoded Polymerase (NEPs), Genome, Pathogens, Plant sciences, Diseases

## Abstract

*Prototheca zopfii* (*P. zopfii*, class Trebouxiophyceae, order Chlorellales, family Chlorellaceae), a non-photosynthetic predominantly free-living unicellular alga, is one of the few pathogens belonging to the plant kingdom. This alga can affect many vertebrate hosts, sustaining systemic infections and diseases such as mastitis in cows. The aim of our work was to sequence and assemble the *P. zopfii* genotype 1 and genotype 2 mitochondrial and plastid genomes. Remarkably, the *P. zopfii* mitochondrial (38 Kb) and plastid (28 Kb) genomes are models of compaction and the smallest known in the Trebouxiophyceae. As expected, the *P. zopfii* genotype 1 and 2 plastid genomes lack all the genes involved in photosynthesis, but, surprisingly, they also lack those coding for RNA polymerases. Our results showed that plastid genes are actively transcribed in *P. zopfii*, which suggests that the missing RNA polymerases are substituted by nuclear-encoded paralogs. The simplified architecture and highly-reduced gene complement of the *P. zopfii* mitochondrial and plastid genomes are closer to those of *P. stagnora* and the achlorophyllous obligate parasite *Helicosporidium* than to those of *P. wickerhamii* or *P. cutis*. This similarity is also supported by maximum likelihood phylogenetic analyses inferences. Overall, the *P. zopfii* sequences reported here, which include nuclear genome drafts for both genotypes, will help provide both a deeper understanding of the evolution of *Prototheca* spp. and insights into the corresponding host/pathogen interactions.

## Introduction

Organisms belonging to the genus *Prototheca* are achlorophyllous algae widespread in the environment. The genus is classified within the class of Trebouxiophyceae, order Chlorellales and family Chlorellaceae, and historically encompasses six species: *P. stagnora*, *P. ulmea*, *P. wickerhamii*, *P. blaschkeae*, *P. zopfii* and *P. cutis*^1–3^. A seventh species, *P. miyajii*, has very recently been isolated in a patient with systemic protothecosis and classified as a separate species due to some genetic and phenotypical differences from *P. wickerhamii*^4^. Finally, an eighth species, *P. moriformis*, is not currently considered a species *per se* because of its biochemical/genetic resemblance to *P. zopfii* and because of its high intraspecific heterogeneity^2,5^.

All *Prototheca* species have forfeited their photosynthetic capabilities, and, consequently, their ability to harvest energy from light and fix carbon, having undergone an evolutionary transition from autotrophy to heterotrophy^3^, favoured also by the ability of some species to sustain infectious diseases in both humans and animals^6,7^. *P. wickerhamii*, *P. cutis* and *P. blaschkeae*, in particular, have been associated with human diseases, especially in the presence of impaired immunological-cellular systems^1,7,8^. Nevertheless, *P. wickerhamii*, *P. blaschkeae* and *P. zopfii* can also infect animals, especially dogs and cows^6,9,10^. Among them, *P. blaschkeae* and *P. zopfii*, are the most important species in the veterinary field because of their ability to sustain bovine mastitis^11–13^. *P. zopfii* can be further divided into two genotypes, namely genotype 1 and 2, both reported as pathogenic for humans^14^, whereas genotype 2 is the most isolated *Prototheca* in bovine mastitis outbreaks worldwide^11,12,15–18^.

The sequences of *Prototheca* species currently available in public databases are those of the 18S rDNA (small subunit of rDNA, SSU) and 28S rDNA (large subunit of rDNA, LSU)^2,19^, and those of the Internal Transcribed Spacer regions (ITS), as well as some mitochondrial and plastid genomes. Notably, this information is not available for all species and, full-length sequences are very often missing^12,20,21^: complete sequences of the organellar DNA are currently only available for *P. wickerhamii* (both mitochondrion and plastid)^22,23^, *P. cutis* and *P. stagnora* (plastid only)^24^.

In this paper, we present the complete and manually annotated genomes of mitochondria and plastids of *P. zopfii* genotypes 1 and 2, and the first draft assembly of the whole nuclear genomes of both *P. zopfii* subspecies. Our work gives, for the first time, a representative overview of the extreme reduction which occurs within the mitochondrial and plastid genomes of these algae and provides basic information for further investigations.

## Materials and Methods

### Strains and culture conditions

*P. zopfii* genotype 1 (SAG 2063) and *P. zopfii* genotype 2 (SAG 2021) were obtained from the Culture Collection of Algae at Göttingen University (“Sammlung von Algenkulturen der Universität Göttingen”, SAG, Göttingen, Germany). The strains were aerobically sub-cultured on Sabouraud Dextrose Agar plates for 48–72 h at 30 °C until DNA isolation was carried out.

### Isolation of DNA and RNA

Genomic DNA and RNA were extracted starting from approximately one g of *P. zopfii* genotype 1 and genotype 2 resuspended in 20 ml wash solution (0.6 M Sucrose, 20 mM Tris, 20 mM MgCl_2_ and 1 mM DTT, pH 7.5) and centrifuged at 2200 g for 2 min at 4 °C.

For DNA, washed pellets were resuspended in 500 μl of TRIS EDTA lysis buffer (10 mM Tris-HCl, 10 mM EDTA, 250 mM NaCl, pH 8), supplemented with 25 µl of proteinase K (20 mg/ml) (Sigma-Aldrich, St. Louis, MO, USA), 25 µl of SDS 10% and incubated at 56 °C for 2 h. Next, 25 µl of RNaseA (20 mg/ml) (Sigma-Aldrich) were added and the suspensions were incubated at 56 °C for 30 min. DNA was extracted using an equal volume of 1:1 (v/v) phenol:chloroform and precipitated with one volume of cold isopropanol. DNA was rinsed with 70% (v/v) cold ethanol, air dried, resuspended in 30 µl of ultrapure water and stored at −20 °C until use. DNA concentration and quality were estimated by PicoGreen (Thermo Fisher, Waltham, MA, USA) and by agarose gel electrophoresis.

Total RNA was extracted from washed pellets with TRIzol (Invitrogen, Carlsbad, CA, USA) and purified by NucleoSpin miRNA kit (Macherey-Nagel, Duren, Germany), following the manufacturer’s protocol, in combination with TRIzol lysis. RNA concentration (ng/µl) and quality RNA Integrity Number (RIN) were determined by Agilent 2100 Bioanalyzer (Santa Clara, CA, USA). RNA extracts were stored at −80 °C until use.

### DNA and RNA library preparation and sequencing

*P. zopfii* DNA libraries for Illumina sequencing (paired-end and mate-pair sequencing) (Illumina, San Diego, CA, USA) were prepared and sequenced on an Illumina MiSeq instrument, following the manufacturer’s instructions as detailed in Supplementary Table 1. DNA library preparation for GS-FLX sequencing was performed using the GS FLX Titanium Rapid Library Preparation Kit (Roche, Basel, Switzerland) as follows: 1 μg DNA for each *P. zopfii* strain was used in the preparation of shotgun libraries by genomic DNA fragmentation by nebulization and ligation to specific adapters. According to the manufacturer’s instructions, libraries were subjected to clonal amplification by emulsion PCR reaction, recovered by isopropanol breaking and enriched for positive reaction beads. Each library was separately loaded onto one region of the GS-FLX PicoTiter Plate and sequenced according to the 454 GS-FLX Titanium XL protocol.

One μg of RNA was used for libraries construction using TruSeq^®^ RNA Sample Preparation v2 Kit (Illumina), according to the manufacturer’s instructions using poly(A) enrichment and sequenced on a 2 × 101-cycles Hiseq 2000 run (Illumina).

### *De novo* genome assembly

The whole genomic sequence collections - composed of Illumina paired-end and mate-pair sequences, plus GS-FLX reads - from *P. zopfii* genotype 1 and 2 were assembled with SPAdes^25^ in read error correction and assembling mode. Six K values, ranging from K21 to K127, were automatically selected by the algorithm based on the read length and dataset type. CAP3^26^ (-p 96 -o 500) was run on the resulting scaffolds to further assemble contiguous regions. Illumina reads were mapped back to scaffolds by means of bwa mem (v. 0.7.10^27^), keeping only those having ≤2 mismatches with the reference and Pilon (v 1.22)^28^ in assembly improvement mode was employed in order to correct substitutions and short indels.

### Organelles assembly

Among the assembled scaffolds, sequences of mitochondrion and plastid were detected by homology with a set of 322 mitochondrial and 605 plastid/chloroplast sequences from related species (i.e.: green algae) and circularized, giving rise to full circular sequences for both organelles in both *P. zopfii* genotypes. Assembly of the mitochondrial and plastid genomes of *P. zopfii* was independently confirmed by a custom assembly strategy that, starting from “seeds”, implements an iterative procedure aimed at finding reads partially overlapping with the seed and assembling them with the original contig. Then, overlaps among the new contigs were used to generate a “supercontig”. Further alignment-assembly-extension steps were performed on each side of the supercontig until an overlap between the 5′ and 3′ ends of the sequence was found, meaning that the whole circular genome had been covered. A full description of this custom assembly procedure is available in Supplementary Methods.

### Nuclear genome annotation

Gene prediction in nuclear genomes was performed with Augustus^29^ using *Chlorella variabilis* as reference species. A double annotation procedure was carried out on the predicted protein models, employing both BLAST^30^ (-evalue 1e^−10^) versus the UniProtKB database (http://www.uniprot.org/uniprot/), and InterProScan 5^31^ to provide functional analysis. BLAST comparison (-evalue 1e^−10^) was also performed between the annotated protein datasets of the two *P. zopfii* genotypes. Annotation of proteins identified as putative DNA-directed RNA polymerases (NEPs) was double-checked by BLASTp against the NCBI’s own non-redundant (“nr”) database. Presence of a chloroplast transit peptide (cTP) in genes annotated as NEPs was assessed by using several prediction tools available on the web: PProwler^32^, PredAlgo^33^, TargetP (v1.1)^34^, and predSL^35^.

### Organelles annotation

Organelles were annotated using the DOGMA webserver^36^. Annotation was manually refined, relying on similarities obtained by BLAST versus the organellar genes of the closest known relatives of *P. zopfii* (i.e.: *C. variabilis*, *Auxenochlorella protothecoides, Helicosporidium* sp. and *P. wickerhamii*). Transfer RNA (tRNA) and ribosomal RNA (rRNA) sequences were determined by BLASTn alignment, whereas coding DNA sequences (CDS) positions were refined on the basis of protein-protein (BLASTp) matches. Circular representations of the mitochondria and plastids were drawn using Gview^37^. Comparison of CDS among species was performed by BLASTp.

### Phylogenetic analysis

The protein sequences inferred from nine conserved plastid ribosomal genes (RPL2, RPL5, RPL14, RPL16, RPL20, RPS8, RPS11, RPS12, RPS14) were retrieved from *P. zopfii* genotype 1 and 2, from related species (i.e.: *C. variabilis*, *A. protothecoides, Helicosporidium* sp., *P. wickerhamii*, *P. cutis* and *P. stagnora*), and from other Trebouxiophyceae species available at NCBI, for a total of 40 species. Only ribosomal proteins whose sequences were available for each considered species were taken into account. Sequences were concatenated and aligned to produce a super-alignment with Clustal-Omega^38^, which was manually inspected and used to infer a Maximum Likelihood phylogenetic tree with the program PhyML^39^ using four substitution rate categories, the cpREV substitution model, estimated gamma shape parameter, 1000 bootstraps, and core Trebouxiophyceae as outgroup.

### RNA-Seq data analysis

RNA-Seq reads from *P. zopfii* 1 and 2 were mapped to the respective draft assemblies with STAR aligner (v 2.5.3a)^40^. Alignments were filtered retaining, for each genotype, only reads aligning for ≥80% of their length and having ≤2 mismatches. The number of reads mapping within the predicted gene models was assessed with BEDtools (v 2.26.0)^41^, requiring a read to map for at least 90% of its length within a gene to be counted. Counts were then converted to reads-per-kilobase-per-million (RPKM) values.

## Results

The genomes of *P. zopfii* genotype 1 and 2 were sequenced using a combination of different approaches (Supplementary Table 1) resulting in 45,166,626 and 66,488,185 total reads, respectively.

### Sequence assembly

Sequence assembly yielded a nuclear genome of 26,448,891 and 24,744,895 bp for *P. zopfii* genotype 1 and genotype 2, respectively; organelles were very small: mitochondria being 38,164 and 39,222 bp for genotypes 1 and 2, respectively, whereas plastids being 28,698 and 28,686 bp, for genotype 1 and 2 respectively (Table 1).

**Table 1:** Organelles and nuclear genome annotation statistics.

		Size (nt)	Number Scaffolds	%GC	Total features	CDS	tRNA	rRNA	Number introns (size)	GenBank Accession
Mitochondrion	*C. variabilis*	78,500	1	28.2	62	32	27	3	6 (5,482)	NC_025413.1
	*A. protothecoides*	57,274	1	28.7	70	39(+2^c^)	26	3	7 (6,589)	NC_026009.1
	*Helicosporidium* sp.	49,343	1	25.6	65	37	25	3	2 (8,208)	NC_017841.1
	*P. wickerhamii*	55,328	1	25.8	65	36	26	3	5 (4,709)	NC_001613.1
	*P. zopfii* gen. 1	38,164	1	28.7	62	33	26	3	0 (0)	MF197533.1
	*P. zopfii* gen. 2	39,222	1	28.7	63	34	26	3	1 (776)	MF197534.1
Plastid	*C. variabilis*	124,793	1	34.0	112	79	30	3	3 (1,657)	NC_015359.1
	*A. protothecoides*	84,576	1	30.8	109	76	30	3	0 (0)	NC_023775.1
	*Helicosporidium* sp.	37,454	1	26.9	54	26	25	3	1 (486)	NC_008100.1
	*P. stagnora*	48,188	1	25.7	56	25 (+3^d^)	25	3	0 (0)	AP018372.1
	*P. cutis*	51,673	1	29.7	72	40	29	3	0 (0)	AP018373.1
	*P. wickerhamii*	55,636	1	31.1	70	40	27	3	0 (0)	KJ001761.1
	*P. zopfii* gen. 1	28,698	1	27.0	47	19	25	3	0 (0)	MF197535.1
	*P. zopfii* gen. 2	28,638	1	26.8	47	19	25	3	0 (0)	MF197536.1
Nuclear	*C. variabilis*	42.2 M	414	67.1	9,780	9,780	—	—	n.a.	ADIC00000000.1
	*A. protothecoides*	22.9 M–32.7 M^a^	113	62.8	7,016	7,014	—	2	n.a.	APJO00000000.1
	*Helicosporidium* sp.	12.4 M	5,666	61.7	6,033	6,033	—	—	n.a.	AYPS00000000.1
	*P. stagnora*	16.9 M	27	71.4	7,041	7,041	—	—	n.a.	BCJY00000000.1
	*P. cutis*	20.0 M	29	60.3	6,884	6,884	—	—	n.a.	BCIH01000000.1
	*P. wickerhamii*	~29.0 M^b^	2,860^b^	n.a.	n.a.	n.a.	n.a.	n.a.	n.a.	—
	*P. zopfii* gen. 1	~26.5 M	6,956	67.3	6,884	6,884	—	—	n.a.	PEIA01000000
	*P. zopfii* gen. 2	~24.7M	4,555	73.5	6,381	6,381	—	—	n.a.	PGFX00000000

For each organism, the nuclear or organellar DNA size, the number of scaffolds in the assembly, the percentage of GC content (%GC), the number of introns and size, the number of genes (Total features), subdivided into: coding sequences (CDS), the number of transfer RNAs (tRNA), and of ribosomal RNAs (rRNA) are reported; last column reports NCBI GenBank Accession number for the assemblies.

^a^Original WGS sequencing (BioProject PRJNA182710, year: 2014) covers 21,856,191 bp, whereas a more recent sequencing (PRJNA362700, year: 2017) estimates a total length of 32,730,026 bp.

^b^As reported in^51^.

^c^*A. protothecoides* mitochondrion also includes 2 pseudogenes.

^d^*P. stagnora* plastid also includes 3 ORFs of unknown function.

### Mitochondrial structure and annotation

**Figure 1:**
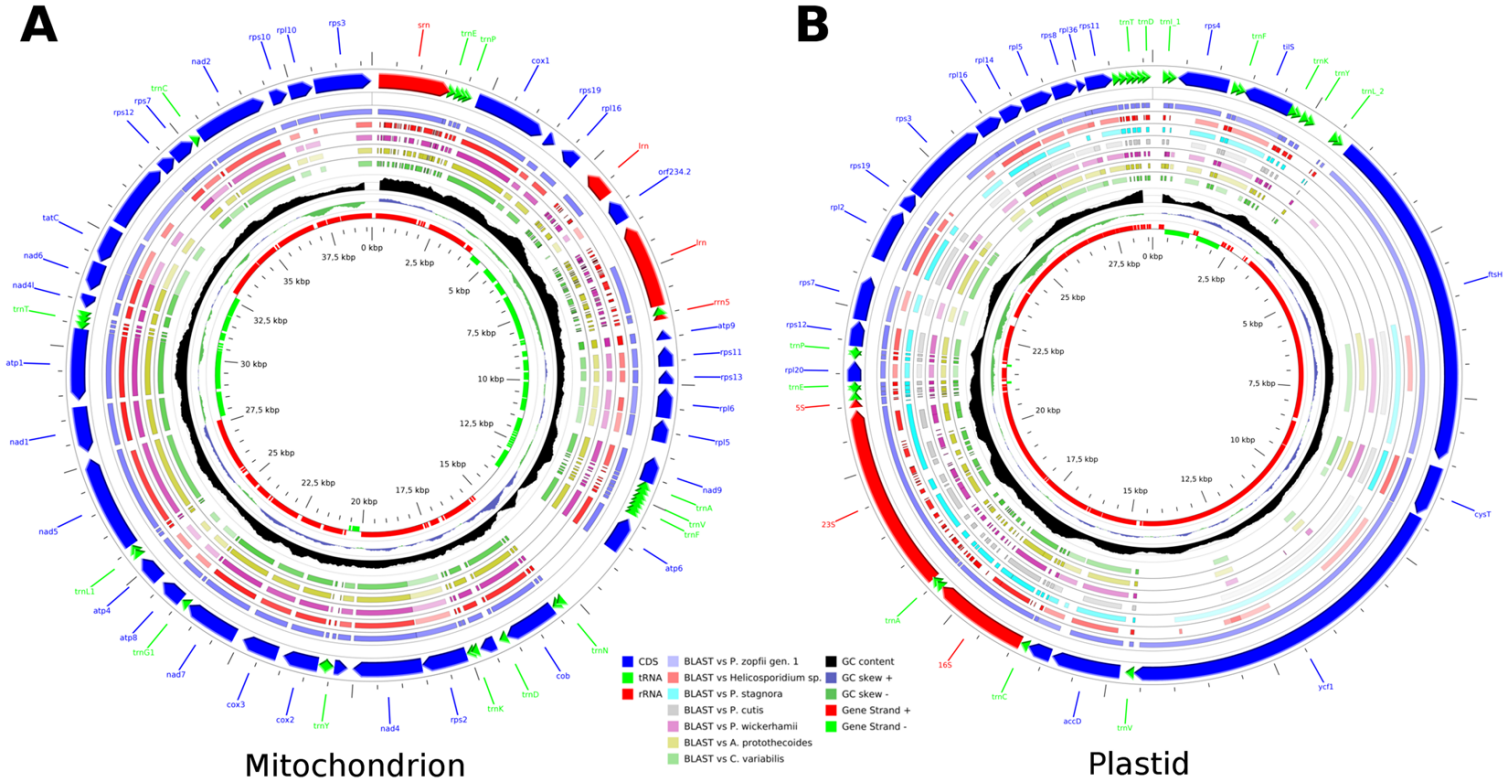
*P. zopfii* genotype 2 mitochondrion and plastid circular plot. Circular plots depicting the annotation of *P. zopfii* genotype 2 mitochondrion (**A**) and plastid (**B**). Gene annotation is reported on the outermost circle of the plot; CDS are in blue, tRNA are in green and rRNA are in red. Innermost circles represent gene orientation, GC content and skew. Other rings report the extent and the % identity of the plastid features with those of proximal organisms (*C. variabilis*, *A. protothecoides*, *Helicosporidium* sp., *P. wickerhamii* plus *P. cutis* and *P. stagnora* for plastid only) and with *P. zopfii* genotype 1. Transparency is proportional to the degree of identity between *P. zopfii* genotype 2 and each reference genome; no transparency indicates 100% identity. % identity was calculated on the basis of BLASTn (for tRNA and rRNA) and BLASTp (for CDS) matches with the corresponding features on the reference plastid genome.

The mitochondrial genomes of both *P. zopfii* genotype 1 and 2 are sized at about 38–39 Kb (Fig. 1A, Supplementary Fig. 1A). They are extremely compact, with only about 32% of non-coding DNA and are characterized by a substantial loss of any intron-exon structure in their genes. Only *P. zopfii* genotype 2 shows a single intron (length: 777 bp) in the long ribosomal subunit (*lrn*) gene, whereas the other species belonging to the Chlorellales (i.e.: *C. variabilis*, *A. protothecoides*, *Helicosporidium* sp., and *P. wickerhamii*) display a more complex structure, with intron length reaching 4,000–8,000 bp. A putative LAGLIDADG homing endonuclease, a class of restriction enzymes directly involved in the DNA cutting process^42^, is encoded within the intron of *lrn* gene.

**Figure 2:**
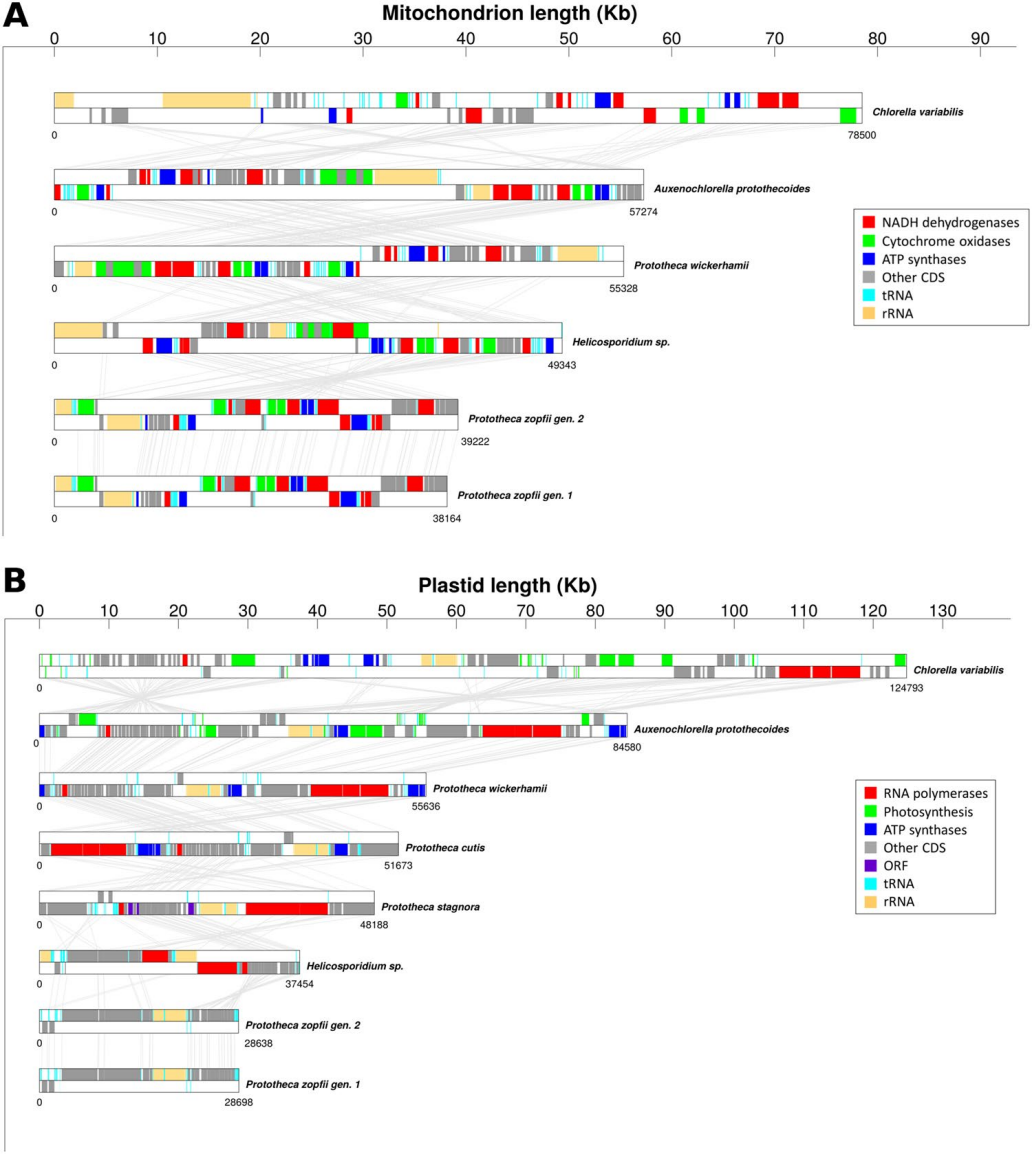
Multi alignment of *P. zopfii* mitochondrion and plastid sequences. Gene order comparison between the mitochondrial (**A**) and plastid (**B**) sequences of *P. zopfii* and other members of the Trebouxiophyceae class. Organisms are ordered by descending organelle size.

The annotation revealed a number of coding genes for both *P. zopfii* species similar to those observed in other Chlorellales species. All the genes encoding for cytochrome units, NADH dehydrogenase, ATP synthases and ribosomal proteins, as well as all the tRNAs and the ribosomal units, have a conserved structure (Table 1, Fig. 2A). As expected, mitochondrial protein similarity between *P. zopfii* genotype 1 and 2 is above 90%, while similarity with *Helicosporidium* sp.*, C. variabilis, A. protothecoides* and *P. wickerhamii* is around 60%.

### Plastid structure and annotation

**Figure 3:**
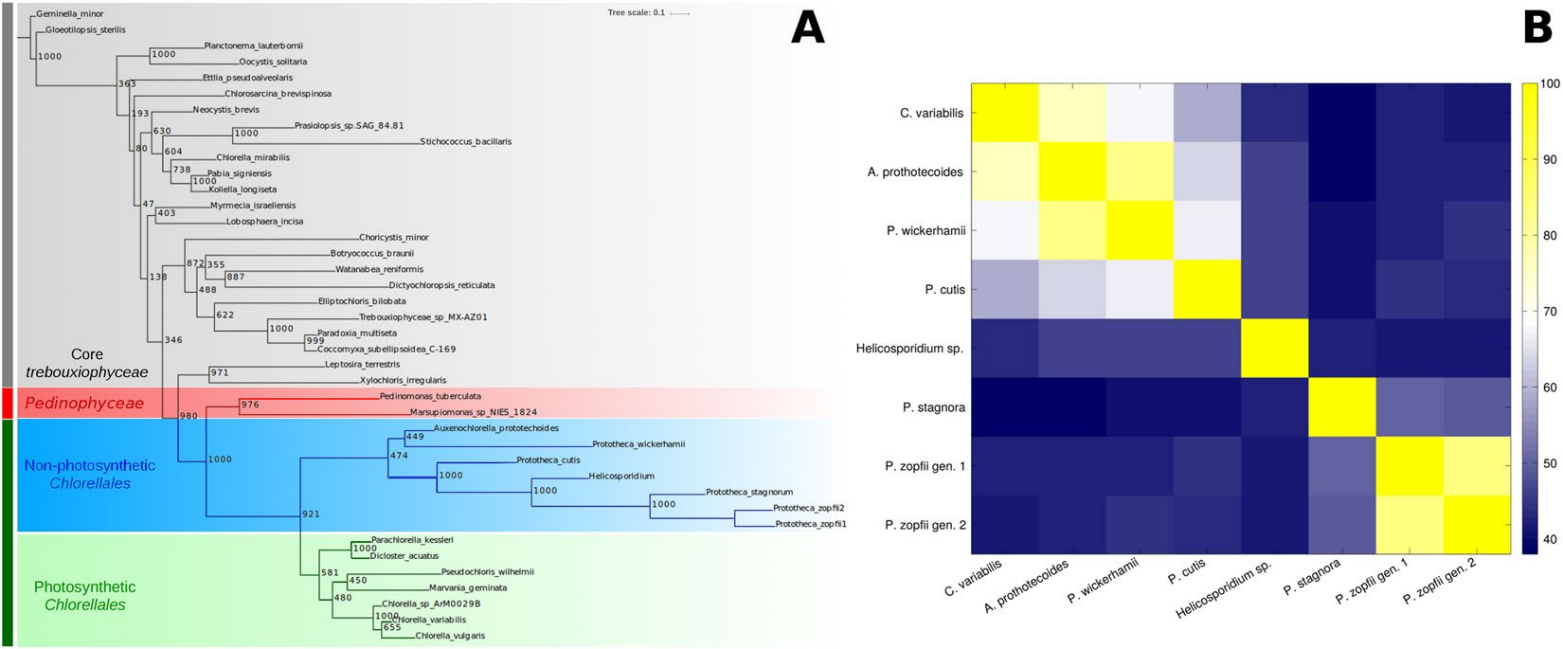
Phylogenetic analysis of *P. zopfii*. (**A**) Maximum Likelihood (ML) tree inferred from the super-alignment of 9 plastid ribosomal proteins (i.e.: RPL2, RPL5, RPL14, RPL16, RPL20, RPS8, RPS11, RPS12, and RPS14). Bootstrap values are indicated above the lines. Core Trebouxiophyceae was used as outgroup. Among Chlorellales, (dark green vertical bar) non-photosynthetic species are highlighted in blue; tree lengths for this group are not drawn to scale. (**B**) Heatmap representing the pairwise average percentage of identity between CDS of 8 organisms belonging to Chlorellales (*P. zopfii* and its closest relatives).

The structure of the plastid genomes of *P. zopfii* genotype 1 and 2 (Fig. 1B, Supplementary Fig. 1B) is very similar to that of other non-photosynthetic algae belonging to the Trebouxiophyceae class, being extremely compact. The genomes are sized only about 28.7 Kb for both genotypes and are the shortest plastid genomes within their class. Both *P. zopfii* genotypes possess only 19 CDS, and as a result are simpler than those of *Helicosporidium* sp. (26 CDS), *P. wickerhamii*, *P. cutis* (both possessing 40 CDS) and *P. stagnora* (28 CDS). On the other hand, the tRNAs and rRNAs are conserved among all these species and other photosynthetic algae (i.e.: *C. variabilis* and *A. protothecoides*) (Table 1, Fig. 2B). Plastid protein similarity between genotype 1 and 2 is about 85% while similarity with other organisms is around 41–43%, with the best match being with *P. stagnora* (i.e.: 49.9% and 49.5% similarity to *P. zopfii* 1 and 2, respectively) (Fig. 3A). Comparison with other related organisms shows that the plastid genomes of *P. zopfii* genotype 1 and 2, like those of *Helicosporidium* sp., *P. wickerhamii*, *P. cutis* and *P. stagnora*, lack the genes associated with photosynthesis (photosystem I and II, chlorophyll biosynthesis and cytochrome components), whereas ATP synthase genes were maintained only in *P. wickerhamii* and *P. cutis*. Ribulose-1,5-bisphosphate carboxylase/oxygenase large subunit (*rbcL*, or RuBisCO) is absent in *P. zopfii*, as well as in all other *Prototheca* spp. and in *Helicosporidium* sp. Moreover, differently to the other Trebouxiophyceae, *P. zopfii* also lacks all plastid-encoded RNA polymerases (i.e.: *rpoA, rpoB, rpoC1* and *rpoC2*) (Table 2, Supplementary Table 2). Our transcriptome analysis demonstrated that most plastid genes encoding rRNA and proteins were expressed in *P. zopfii* (Supplementary Fig. 2A), although three of them (i.e.: rps4, rps7, rpl20) had low RPKM values, suggesting that nuclear-encoded counterparts could compensate for the loss of plastid-encoded RNA polymerases.

**Table 2:** Comparison of genes of the *C. variabilis* chloroplast with genes of the *P. zopfii* and *P. stagnora* plastids.

Function^a^		Present^b^	Absent^c^
Transcription (plastid-encoded RNA polymerase)		—	**rpoA**, **rpoB**, **rpoC1**, **rpoC2**
Translation		—	**tufA**
Ribosomal proteins	Small subunit	rps3, rps4, rps7, rps8, rps9, rps11, rps12, rps14, rps19	rps2, rps18
	Large subunit	rpl2, rpl5, rpl14, rpl16, rpl20, **rpl36**	rpl12, **rpl19**, rpl23, **rpl32**
Photosynthesis	ATP synthase	—	atpA, atpB, atpE, atpF, atpH, atpI
	Photosystem I	—	psaA, psaB, psaC, psaI, psaJ, psaM
	Photosystem II	—	psbA, psbB, psbC, psbD, psbE, psbF, psbH, psbI, psbJ, psbK, psbL, psbM, psbN, psbT, psbZ
	Cytochromecomplex	—	petA, petB, petD, petG, petL
Metabolism		accD, cysT	rbcL, cysA
Chlorophyll biosynthesis		—	chlB, chlI, chlL, chlN
Protein quality control		**ftsH**	clpP
Assembly, membrane insertion		—	ccsA, secG

Genes are grouped according to function. ^a^Function and names of chloroplast genes follow the categorization and nomenclature reported in^52^.

^b^Genes present in *P. zopfii*. Genes present in *P. zopfii* genotype 1 and 2 but absent in *P. stagnora* plastid are in boldface.

^c^Genes absent in both *P. zopfii* and *P. stagnora*. Genes present in *P. stagnora* but absent in *P. zopfii* genotype 1 and 2 plastid are in boldface.

Both *P. zopfii* genotypes plastids contain the genes of most of the ribosomal proteins, with the exception of RPL12, RPL19, RPL23, RPL32, RPS2, RPS18 and RPS9. The same proteins, with the exception of RPL19 and RPL32, are also absent in *P. stagnora*, which, apparently, is the most similar organism. The phylogenetic tree inferred from the super-alignment of 9 shared ribosomal proteins confirms that *Helicosporidium* sp., *P. stagnora* and *P. zopfii* are closely related genera, whereas *P. wickerhamii* is more distant, closer to *A. protothecoides* than to other *Prototheca* species (Fig. 3B).

### Nuclear genome annotation

From the genome assembly procedure, 6,956 and 4,555 scaffolds with lengths >1 Kb representing the nuclear genome were obtained for P*. zopfii* genotype 1 and 2, respectively, indicating a genome size of about 26.5 Mbp and 24.7 Mbp for genotypes 1 and 2, respectively, a size between those of *Helicosporidium* sp. (12.4 Mb) and *C. variabilis* (46.2 Mb) and comparable to the genome of *A. protothecoides* (~23 Mb) and *P. wickerhamii* (~29 Mb) (Table 1).

The maximum scaffold length was 97,625 and 57,068 bp, with 1,289 and 1,708 contigs exceeding 5 Kb in length and a N50 value of 6,686 and 7,940 for *P. zopfii* genotypes 1 and 2, respectively.

Augustus gene prediction led to the individuation of 6,884 and 6,381 gene models for the two genotypes. 56.5% and 62.0% of *P. zopfii* genotype 1 gene models were annotated versus UniProtKB and InterPro databases, respectively, while the corresponding percentages for genotype 2 were 59.4% and 67.2%. The main features of the nuclear genome, such as gene number and coding density, were consistent for both genotypes, and similar to their counterparts in *A. protothecoides* genome (i.e.: gene density: 0.26 genes/Kb and 0.32 genes/Kb, for both *P. zopfii* genotypes and *A. protothecoides*, respectively). On the contrary, the average exon and intron sizes were higher compared to those previously observed in related species (Supplementary Table 3). BLAST comparison of the predicted proteins of the two *P. zopfii* genotypes resulted in a set of 6,134 common entities, representing a core set of homologous proteins conserved in the two genotypes. Among the predicted genes and transcripts, we found evidence of the presence and expression of nuclear-encoded polymerases (NEPs) (Supplementary Data 1 and 2). *P. zopfii* genotype 1 and 2 possess 21 and 19 genes annotated as NEPs, respectively, and both showed a RNA-Seq signal indicating active transcription of many of them (Supplementary Fig. 2B,C). Prediction of target peptides highlighted at least one NEP per genotype as a high-confidence candidate for containing a chloroplast transit peptide (cTP) (genes g5108 and g2780 for genotypes 1 and 2, respectively, for which all the four prediction software employed were concordant); moreover, PredAlgo also suggested two more genes (g3914 and g4216 for genotypes 1 and 2, respectively) to be plastid-directed NEPs. In addition to that, we found evidence of some mitochondrial targeting peptides (mTPs) in more gene models (Supplementary Table 4).

## Discussion

In this paper, we describe the complete, manually annotated, circular sequence of both mitochondrial and plastid organellar DNA of *P. zopfii* genotype 1 and genotype 2, as well as a first draft of the complete genome, by whole genome shotgun sequencing.

Structure of the mitochondrial genomes of both *P. zopfii* genotypes was revealed to be smaller in size and extremely condensed when compared to that of some related organisms, i.e.: *P. wickerhamii*^22^ and *Helicosporidium* sp.^43^, but similarly functional, with the size reduction mostly due to the lack of intron-exon structures.

An extremely compact and simplified structure was also observed in *P. zopfii* plastid genomes, which showed a substantially reduced size (about 28.7 Kb), smaller than those of all other algae belonging to the class of Trebouxiophyceae. As previously observed in the genomes of non-photosynthetic algae belonging to this class, *P. zopfii* plastids lack all the genes for the synthesis of the proteins involved in the photosynthesis process^23,44^, and for the RuBisCO large subunit. Fundamental plastid-related functions, however, seem to have been preserved, as indicated by the presence of a RNA-Seq signal on the genes of plastid-encoded ribosomal proteins. The low expression of some of them, however, cannot preclude their presence in the genome as pseudogenes in *P. zopfii*. However, further experiments should be carried on in order to confirm this observation. More interestingly, the entire set of *rpo* genes (i.e. *rpoA*, *rpoB*, *rpoC1* and *rpoC2*), which codify for the plastid-encoded RNA polymerases (PEPs), was lost in *P. zopfii*, an unprecedented observation within this class of algae. Loss of PEPs was previously reported for other non-photosynthetic parasitic plants, such as *Cuscuta obtusiflora*^45^ and *Rhizanthella gardneri*^46^, but not in apicomplexan and algae: plastid genomes of *Plasmodium falciparum*^47^, *A. protothecoides*, *P. wickerhamii*^23^, *P. cutis*, *P. stagnora*^24^ and *Helicosporidium* sp.^44^ have all retained the complete set of *rpo* genes. We found no evidence of PEP sequences either in plastid assemblies or in the nuclear draft genomes, whereas nuclear genome contigs contained evidence of 21 and 19 DNA-driven, nuclear-encoded polymerases (NEPs) for *P. zopfii* genotype 1 and 2, respectively, and at least one of them, per genotype, was predicted to contain plastid-targeting signal peptides. It is therefore possible that *P. zopfii* codes for other NEPs able to target the plastid, making it possible to transcribe its genetic information.

As previously suggested^23,24,48,49^, considering the degree of similarity between the few structural genes preserved in its plastid genome and the evidence from the phylogenetic analysis, *P. zopfii* seems to be more closely related to *P. stagnora* and to *Helicosporidium* sp., rather than to *P. wickerhamii* or *P. cutis*. Moreover, it is noteworthy that *P. wickerhamii* appears not to be closely related to *P. zopfii*, but instead to *A. protothecoides*, strengthening the evidence that *P. wickerhamii* is only loosely related to other *Prototheca* spp, as previously revealed by plastid genome comparison^23^ and supporting the proposal of either moving *P. wickerhamii* into *Auxenochlorella* genus or creating a new genus^48^.

Nuclear genome assemblies of *P. zopfii* genotype 1 and 2 had a size estimated at about 25–26 Mb for both, consistent with that reported in a previous work^50^. Although further studies are certainly needed to elucidate the structure of nuclear genomes of *P. zopfii*, this work adds information to the growing body of genome resources for the plant kingdom, being, although a preliminary draft, the first report of the assembly of nuclear DNA of *P. zopfii*.

In conclusion, we believe that the information reported herein will be important for the understanding of the evolution and genomic organization of *Prototheca* spp., with a particular focus on the progressive loss of functions of plastids in the shift from autotrophic, photosynthetic, to obligate, heterotrophic, parasitic algae.

## Electronic supplementary material

Supplementary information

## Data Availability

The complete genome sequencing project has been registered in the NCBI BioProject portal (https://www.ncbi.nlm.nih.gov/bioproject/) under the accession number PRJNA388740. Raw DNA sequence reads for *P. zopfii* genotypes 1 and 2 have been deposited into the NCBI Short-Read Archive (SRA, https://www.ncbi.nlm.nih.gov/sra/) under the accession numbers SRR6319956-SRR6319964. RNA-seq reads are saved under the accession numbers SRR7091517-SRR7091518. Full sequences of mitochondria and plastids are available in GenBank, under accessions MF197533, MF197534, MF197535, and MF197536. The Whole Genome Shotgun project has been deposited (as non-annotated contigs) at DDBJ/ENA/GenBank under the accessions PEIA00000000 and PGFX00000000. The versions described in this paper are PEIA01000000 and PGFX01000000. Sequences annotated as nuclear-encoded polymerases (NEPs) are available as amino acid FASTA files in Supplementary Data 1 and 2.
