# Author Correction: Genome sequencing of *Prototheca zopfii* genotypes 1 and 2 provides evidence of a severe reduction in organellar genomes

**DOI:** 10.1038/s41598-022-09754-0

**Published:** 2022-04-11

**Authors:** Marco Severgnini, Barbara Lazzari, Emanuele Capra, Stefania Chessa, Mario Luini, Roberta Bordoni, Bianca Castiglioni, Matteo Ricchi, Paola Cremonesi

**Affiliations:** 1 Institute of Biomedical Technologies, National Research Council (ITB-CNR), Segrate, Milan, Italy; 2 PTP-Science Park, Lodi, Italy; 3 Institute of Agricultural Biology and Biotechnology, National Research Council (IBBA-CNR), Lodi, Italy; 4 Lombardy and Emilia Romagna Experimental Zootechnic Institute (IZSLER), Lodi, Italy; 5 Lombardy and Emilia Romagna Experimental Zootechnic Institute (IZSLER), Piacenza, Italy

Correction to: *Scientific Reports* 10.1038/s41598-018-32992-0, published online 02 October 2018

The original version of this Article contained errors in the Materials and Methods section where the donation of the *P. zopfii* genotype 2 (SAG 2021) was incorrectly attributed to Dr. Jagielski, University of Warsaw (Poland).

In the Materials and Methods section, under the subheading ‘Strains and culture conditions’,

“*P. zopfii* genotype 1 (SAG 2063) was obtained from the Culture Collection of Algae at Göttingen University (“Sammlung von Algenkulturen der Universität Göttingen”, SAG, Göttingen, Germany). *P. zopfii* genotype 2 (SAG 2021) was kindly donated by Dr. Jagielski, University of Warsaw (Poland).”

now reads:

“*P. zopfii* genotype 1 (SAG 2063) and *P. zopfii* genotype 2 (SAG 2021) were obtained from the Culture Collection of Algae at Göttingen University (“Sammlung von Algenkulturen der Universität Göttingen”, SAG, Göttingen, Germany).”

