# Supplementary material for: Genome sequencing of Prototheca zopfii genotypes 1 and 2 provides evidence of a severe reduction in organellar genomes
Source: Sci Rep. 2018 Oct 2;8:14637. doi: 10.1038/s41598-018-32992-0 (PMC6168571; doi:10.1038/s41598-018-32992-0)
Supplement: Supplementary file 1 — Supplementary information [file 41598_2018_32992_MOESM1_ESM.pdf]

Supplementary Information

Genome sequencing of *Prototheca zopfii* 1 and 2 genotypes provides evidence of a severe reduction in organellar genomes

Marco Severgnini[1#] , Barbara Lazzari[2,3#] , Emanuele Capra[3#] , Stefania Chessa[3] , Mario Luini[4] , Roberta Bordoni[1] , Bianca Castiglioni[3] , Matteo Ricchi[*5] , Paola Cremonesi[3]

[1]Institute of Biomedical Technologies, National Research Council (ITB-CNR), Segrate, Milan, Italy

[2] PTP-Science Park, Lodi, Italy

[3] Institute of Agricultural Biology and Biotechnology, National Research Council (IBBA-CNR), Lodi, Italy

[4] Lombardy and Emilia Romagna Experimental Zootechnic Institute (IZSLER), Lodi, Italy

[5] Lombardy and Emilia Romagna Experimental Zootechnic Institute (IZSLER), Piacenza, Italy

[#]These authors equally contributed to this work

Corresponding author: Matteo Ricchi (matteo.ricchi@izsler.it)

SUPPLEMENTARY METHODS **3**
SUPPLEMENTARY REFERENCES 6
SUPPLEMENTARY FIGURES **7**
SUPPLEMENTARY FIGURE 1 8
SUPPLEMENTARY FIGURE 2 10
SUPPLEMENTARY TABLES **11**
SUPPLEMENTARY TABLE 1 12
SUPPLEMENTARY TABLE 2 13
SUPPLEMENTARY TABLE 3 17
SUPPLEMENTARY TABLE 4 18
SUPPLEMENTARY DATA **19**
SUPPLEMENTARY DATA 1 20
SUPPLEMENTARY DATA 2 26

Supplementary Methods

The iterative assembly strategy devised to independently confirm the assemblies of the mitochondrial and plastid genome of both *P. zopfii* genotype 1 and *P. zopfii* genotype 2 was inspired by Enly **[SR1]**, a tool based on the iterative mapping of sequence reads at contig edges, originally implemented for Newbler-like assemblies (i.e.: reads from Roche 454 pyrosequencing) and by IMAGE **[SR2],** which uses Illumina short-read sequences to improve draft genome assemblies by aligning sequences against contig ends and performing local assemblies to produce gap-spanning contigs.

The procedure is mainly constituted by 5 different steps:

1. Determination of assembly seeds
2. Extension of the assembly from each seed
3. Find overlaps among the extended seeds and obtain a "supercontig"
4. Extension of the supercontig on 5'- and 3'- end
5. Verification of circularization

1. Determination of assembly seeds

The first step of the custom assembly procedure was the search for proper "seeds" to start from the process of progressive assembly of the reads to the contigs. To this aim, we, firstly mapped all the raw reads obtained from the genomic sequencing to a proper reference, chosen among those of closely related species in the Trebouxiophyceae class. As a reasonable reference, we chose *P. wickerhamii* mitochondrion (accession: NC_001613.1) and plastid (accession: KJ001761) sequences. Reads were aligned to the reference using bwa mem (v 0.7.2, **[SR3]**) and optical duplicates were removed by Picard tools (v 1.115, http://broadinstitute.github.io/picard). Seeds were defined as contiguous stretches of bases mapped at high-depth on the reference genome by *P. zopfii* reads. Threshold for "high-depth" was chosen empirically to 1000× for the mitochondrion and to 100× for the plastid.

2. Extension of the assembly from each seed

For each of the initial seeds, the procedure enters a loop of repeated assembly-mapping steps aimed at finding reads partially overlapping with the seed and assembling them with the original contig. The procedure performs the following operations: mapping of the initial reads on the reference; assembly by Abyss (v. 1.9.0, k-mer selected: 64, **[SR4]**); check whether the newly

obtained contig is longer than the original one; set this extended contig as new reference; re-perform the mapping against the new reference. The procedure is iterated as long as the generated contig is longer than the one obtained by the previous step.

3. Find overlaps among the extended seeds and obtain a "supercontig"
After all the seeds have been extended as described in step 2, overlaps among the new contigs are assessed and a "supercontig" is generated by assembling all the overlapping sequences by phrap (v. 1.09, **[SR5]**). The initial reads are, then, mapped on this new reference.

4. Extension of the supercontig on 5'- and 3'- end
Further read alignment-assembly-extension steps are performed following the same procedure as described in step 2, with some minor variants. Briefly, for each extension step, we tested many combinations of the "-k" parameter of Abyss assembler, in order to get the best possible assembly (i.e.: the one generating only one, longer, contig) for each step; moreover, for limiting the number of reads to be assembled in each step, we select only those reads mapping within a 200 bp span from the contig edge; finally, if the Abyss assembly does not generate a single contig, we perform another assembly of the reads using phrap. In case this latter does not result in a single contig, the longer contig from the assembly is chosen. This procedure is iterated until the newly-generated contig is longer than the reference at the previous step and it is repeated for both the 5' and 3' ends until an overlapping between the sequence at the ends of the supercontig is found, meaning that the whole mitochondrial or plastid genome is covered.

5. Verification of circularization
In order to verify the actual circularization of the sequence, we align the final contig against itself by Blat **[SR6]** and check a partial overlap between the 5'- and 3'-ends of the reference. Moreover, mapping all the reads against the new reference, we isolate all the read pairs showing an "abnormal" mate distance (i.e.: >1000 bp). These pairs result always mapping at the edges of the reference, showing that, in reality, they are placed on adjacent regions of the circular chromosome.

In this way we were able to independently assemble the mitochondrion and the plastid genome of both *P. zopfii* genotype 1 and 2. The sequence for all these assemblies results equivalent to that

obtained by the Spades-based **[SR7]** assembly.

Supplementary references

[SR3] Li H. (2013) Aligning sequence reads, clone sequences and assembly contigs with BWA-MEM. arXiv:1303.3997v1 [q-bio.GN].

[SR2] Tsai IJ, Otto TD, Berriman M. Improving draft assemblies by iterative mapping and assembly of short reads to eliminate gaps. Genome Biol. 2010;11(4):R41. doi: 10.1186/gb-2010-11-4-r41. PubMed PMID: 20388197; PubMed Central PMCID: PMC2884544.

[SR1] Fondi M, Orlandini V, Corti G, Severgnini M, Galardini M, Pietrelli A, Fuligni F, Iacono M, Rizzi E, De Bellis G, Fani R. Enly: Improving Draft Genomes through Reads Recycling. J Genomics. 2014 Apr 5;2:89-93. doi: 10.7150/jgen.7298. PubMed PMID: 25031660; PubMed Central PMCID: PMC4091449.

[SR4] Simpson JT, Wong K, Jackman SD, Schein JE, Jones SJ, Birol I. ABySS: a parallel assembler for short read sequence data. Genome Res. 2009 Jun;19(6):1117-23. doi: 10.1101/gr.089532.108. PubMed PMID: 19251739; PubMed Central PMCID: PMC2694472.

[SR5] de la Bastide M, McCombie WR. Assembling genomic DNA sequences with PHRAP. Curr Protoc Bioinformatics. 2007 Mar; Chapter 11:Unit11.4. doi: 10.1002/0471250953.bi1104s17. PubMed PMID: 18428783.

[SR6] Kent WJ. BLAT--the BLAST-like alignment tool. Genome Res. 2002 Apr;12(4):656-64. PubMed PMID: 11932250; PubMed Central PMCID: PMC187518.

[SR7] Bankevich A, Nurk S, Antipov D, Gurevich AA, Dvorkin M, Kulikov AS, Lesin VM, Nikolenko SI, Pham S, Prjibelski AD, Pyshkin AV, Sirotkin AV, Vyahhi N, Tesler G, Alekseyev MA, Pevzner PA. SPAdes: a new genome assembly algorithm and its applications to single-cell sequencing. J Comput Biol. 2012 May;19(5):455-77. doi: 10.1089/cmb.2012.0021. PubMed PMID: 22506599; PubMed Central PMCID: PMC3342519.

Supplementary Figures

Supplementary Figure 1. Circular plots depicting the annotation of *P. zopfii* genotype 1 mitochondrion (A) and plastid (B). Gene annotation is reported on the outermost circle of the plot; CDS are in blue, tRNA are in green and rRNA are in red. Innermost circles represent gene orientation, GC content and skew. Other rings report the extent and the % identity of the plastid features with those of proximal organisms (*C. variabilis*, *A. protothecoides*, *Helicosporidium* sp., *P. wickerhamii* plus *P. cutis* and *P. stagnora* for plastid only) and with *P. zopfii* genotype 2. Transparency is proportional to the degree of identity between *P. zopfii* genotype 1 and each reference genome; no transparency indicates 100% identity. % identity was calculated on the basis of BLASTn matches (for tRNA and rRNA) and BLASTp (for CDS) with the corresponding features on the reference plastid genome.

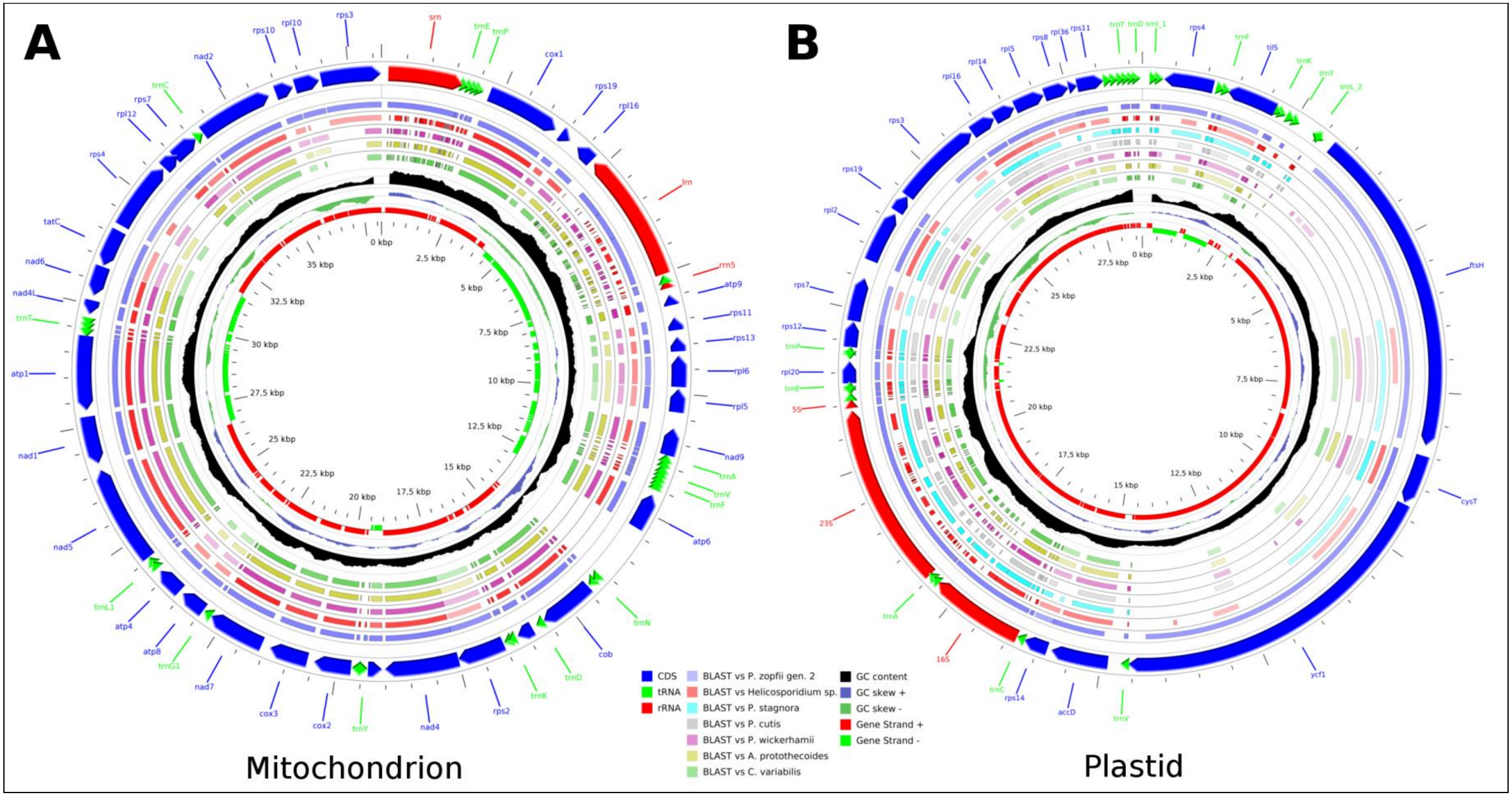

Supplementary Figure 2. Heatmap representing expression values of plastid genes (CDS and rRNA) for *P. zopfii* genotype 1 and 2 (A), candidate NEPs for *P. zopfii* genotype 1 (B, n=21) and genotype 2 (n=19, C). RNA-Seq data are represented as $\log_2$(RPKM).

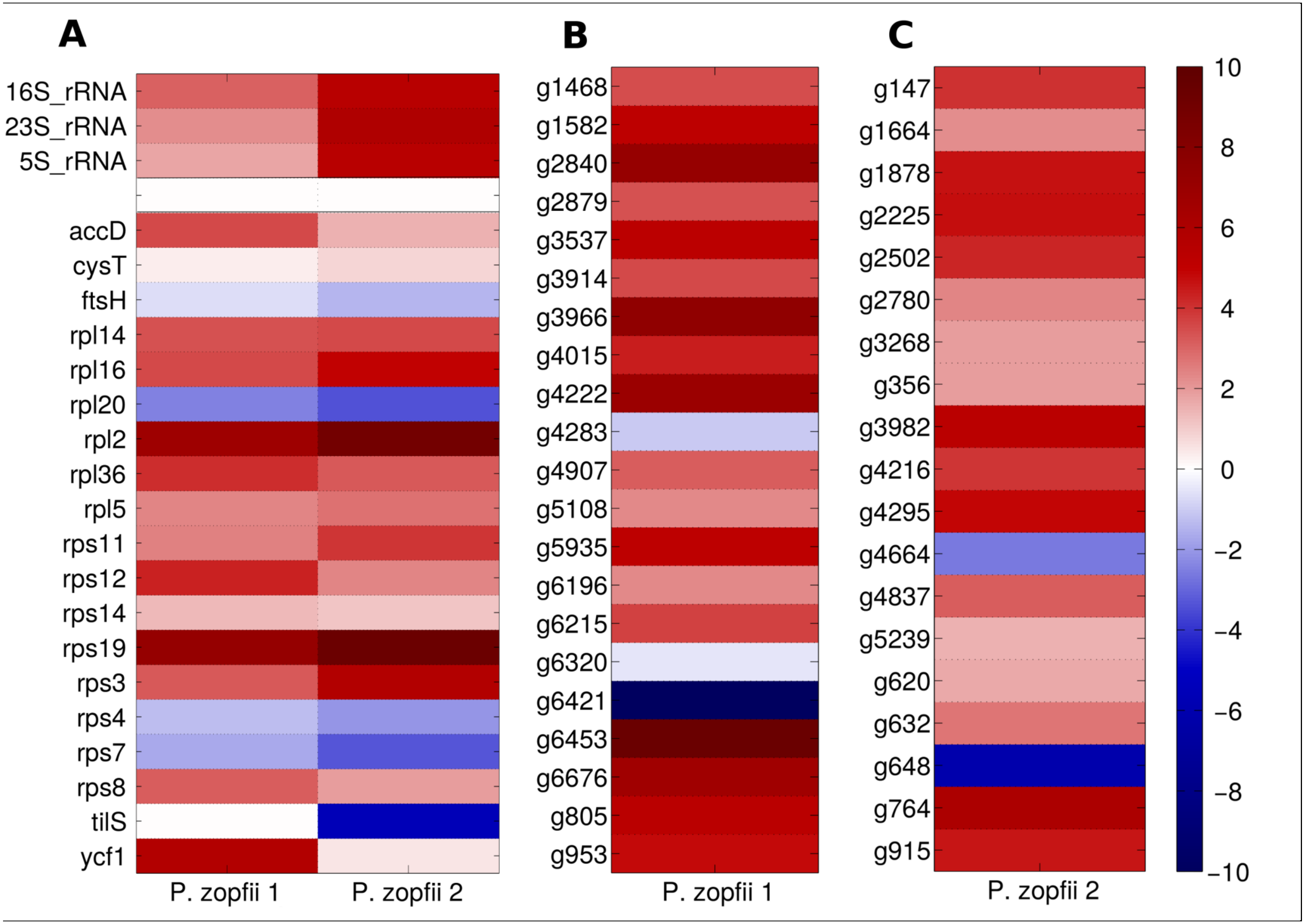

Supplementary Tables

Supplementary Table 1. Reads obtained from NGS sequencing of *P. zopfii* genotype 1 and 2 for both DNA-Seq and RNA-Seq data.

	Species	Strain	Library prep. Kit	Platform	Read type	Nr. of pairs	Nr of Reads
DNA	*P. zopfii* gen. 1	SAG2063	Truseq DNA nano	Miseq	2×300	6,202,135	12,404,270
			Nextera XT	Miseq	2×150	6,892,140	13,784,280
			Nextera mate pair	Miseq	2×300	9,315,103	18,630,206
			Titanium Rapid	454	PLUS	-	347,870
	P. zopfii gen. 2	SAG 2021	Truseq DNA nano	Miseq	2×300	9,556,612	19,113,224
			Nextera XT	Miseq	2×250	10,281,382	20,562,764
			Nextera XT	Miseq	2×150	6,490,523	12,981,046
			Nextera mate pair	Miseq	2×300	6,828,023	13,656,046
			Titanium Rapid	454	PLUS	-	175,105
RNA	*P. zopfii* gen. 1	SAG2063	TruSeq RNA	Hiseq 2000	2×101	55,079,413	110,158,826
	P. zopfii gen. 2	SAG 2021	TruSeq RNA	Hiseq 2000	2×101	60,101,987	120,203,974

Supplementary Table 2. Table reporting the complete chloroplast annotation for *Chlorella variabilis* (C), *Auxenochlorella protothecoides* (A), *Helicosporidium* sp. (H), *Prototheca wickerhamii* (PW), *P. cutis* (PC), *P. stagnora* (PS), *P. zopfii* genotype 1 (PZ1) and *P. zopfii* genotype 2 (PZ2). Black dots represent conserved genes, whereas white circles indicate missing ones. Nomenclature of the genes follows that of *C. variabilis* chloroplast. Function of chloroplast genes follows the categorization reported by Green BR, (Green BR, Plant J., 2011, 66(1), 34-44. doi: 10.1111/j.1365-313X.2011.04541.x)

Category		Gene	Description	Function	C	A	PW	PC	PS[a]	H	PZ1	PZ2
Metabolism		accD	acetyl CoA reductase	--	●	●	●	●	●	●	●	●
		cysT	sulfate transport protein	--	●	●	●	●	●	●	●	●
		cysA	Sulfate/thiosulfate import ATP-binding protein CysA	--	●	○	○	○	○	○	○	○
		rbcL	ribulose-1,5-bisphosphate carboxylase/oxygenase large subunit	RUBISCO subunit: photosynthetic carbon fixation	●	●	○	○	○	○	○	○
Photosynthesis	ATP synthase	atpA	CF1 alpha subunit of ATP synthase	ATP synthesis	●	●	●	●	○	○	○	○
		atpB	beta subunit of ATP synthase	ATP synthesis	●	●	●	●	○	○	○	○
		atpE	CF1 epsilon subunit of ATP synthase	ATP synthesis	●	●	●	●	○	○	○	○
		atpF	CF0 subunit I of ATP synthase	ATP synthesis	●	●	●	●	○	○	○	○
		atpH	CF0 subunit III of ATP synthase	ATP synthesis	●	●	●	●	○	○	○	○
		atpI	CF0 subunit IV of ATP synthase	ATP synthesis	●	●	●	●	○	○	○	○
		minD	MinD cell division protein	septum-site determining protein (membrane-associated ATPase)	●	●	●	●	○	○	○	○
	Cytochrome complex	petA	cytochrome f	electron transport	●	●	○	○	○	○	○	○
		petB	apocytochrome b6 of cytochrome b6/f complex	electron transport	●	●	○	○	○	○	○	○
		petD	cytochrome b6/f complex subunit IV	electron transport	●	●	○	○	○	○	○	○
		petG	subunit V of cytochrome b6/f complex	electron transport	●	●	○	○	○	○	○	○
		petL	subunit VI of cytochrome b6/f complex	electron transport	●	●	○	○	○	○	○	○
	Photosystem I	psaA	P700 apoprotein A1 of photosystem I	photosystem I components: electron transport	●	●	○	○	○	○	○	○
		psaB	P700 apoprotein A2 of photosystem I	photosystem I components: electron transport	●	●	○	○	○	○	○	○
		psaC	Photosystem I iron-sulfur center	photosystem I components: electron transport	●	●	○	○	○	○	○	○
		psaI	photosystem I subunit VIII	photosystem I components: electron transport	●	●	○	○	○	○	○	○
		psaJ	subunit IX of photosystem I	photosystem I components: electron transport	●	●	○	○	○	○	○	○
		psaM	M polypeptide of photosystem I	photosystem I components: electron transport	●	●	○	○	○	○	○	○
		ycf3	photosystem I assembly protein Ycf3	photosystem I assembly protein	●	●	○	○	○	○	○	○
		ycf4	photosystem I assembly protein Ycf4	photosystem I assembly protein	●	●	○	○	○	○	○	○

	Photosystem II	psbA	D1 reaction center protein of photosystem II	photosystem II components: electron transport	●	●	○	○	○	○	○	○
		psbB	photosystem II P680 chlorophyll A apoprotein	photosystem II components: electron transport	●	●	○	○	○	○	○	○
		psbC	CP43 chlorophyll apoprotein of photosystem II	photosystem II components: electron transport	●	●	○	○	○	○	○	○
		psbD	photosystem II protein D2	photosystem II components: electron transport	●	●	○	○	○	○	○	○
		psbE	cytochrome b559 alpha subunit of photosystem II	photosystem II components: electron transport	●	●	○	○	○	○	○	○
		psbF	cytochrome b559 beta subunit of photosystem II	photosystem II components: electron transport	●	●	○	○	○	○	○	○
		psbH	phosphoprotein of photosystem II	photosystem II components: electron transport	●	●	○	○	○	○	○	○
		psbI	I polypeptide of photosystem II	photosystem II components: electron transport	●	●	○	○	○	○	○	○
		psbJ	J protein of photosystem II	photosystem II components: electron transport	●	●	○	○	○	○	○	○
		psbK	PSII K protein	photosystem II components: electron transport	●	●	○	○	○	○	○	○
		psbL	L protein of photosystem II	photosystem II components: electron transport	●	●	○	○	○	○	○	○
		psbM	M protein of photosystem II	photosystem II components: electron transport	●	●	○	○	○	○	○	○
		psbN	N protein of photosystem II	photosystem II components: electron transport	●	●	○	○	○	○	○	○
		psbT	T protein of photosystem II	photosystem II components: electron transport	●	●	○	○	○	○	○	○
		psbZ	Z protein of photosystem II	photosystem II components: electron transport	●	●	○	○	○	○	○	○
		ycf12	Photosystem II reaction center protein	Photosystem II reaction center protein	●	●	○	○	○	○	○	○
	NA	ycf20	Hypothetical chloroplast RF20	plant and algal chloroplasts. As the family is exclusively found in phototrophic organisms it may therefore play a role in photosynthesis (http://www.ebi.ac.uk/interpro/entry/IPR007572)	●	○	○	●	○	○	○	○
Assembly, membrane insertion		ccsA	heme attachment to plastid cytochrome c	biogenesis of c-type cytochromes	●	●	○	○	○	○	○	○
		ycf47	hypothetical chloroplast RF47	Preprotein translocase SecG subunit	●	○	○	○	○	○	○	○

		cemA	chloroplast envelope membrane protein	proton extrusion.<br>Indirectly promotes efficient inorganic carbon uptake into chloroplasts	●	●	○	○	○	○	○	○
		ycf1	ycf1	Integral component of membrane	●	●	●	●	●	●	●	●
		tilS	tRNA(Ile) lysidine synthetase	Integral component of membrane	●	●	●	●	●	●	●	●
Chlorophyll biosynthesis		chlB	ChlB subunit of protochlorophyllide reductase	chlorophyll biosynthesis	●	●	○	○	○	○	○	○
		chlI	protochlorophyllid reductase	chlorophyll biosynthesis	●	●	○	○	○	○	○	○
		chlL	protochlorophyllid reductase	chlorophyll biosynthesis	●	●	○	○	○	○	○	○
		chlN	protochlorophyllid reductase	chlorophyll biosynthesis	●	●	○	○	○	○	○	○
Protein quality control		clpP	proteolytic subunit 2 of clp protease	--	●	●	●	●	○	○	○	○
		ftsH	cell division protein	--	●	●	●	●	○	●	●	●
Ribosomal proteins	Large subunit	rpl12	ribosomal protein L12	large and small subunits of ribosomal proteins - protein synthesis	●	●	●	●	○	●	○	○
		rpl14	ribosomal protein L14	large and small subunits of ribosomal proteins - protein synthesis	●	●	●	●	●	●	●	●
		rpl16	ribosomal protein L16	large and small subunits of ribosomal proteins - protein synthesis	●	●	●	●	●	●	●	●
		rpl19	ribosomal protein L19	large and small subunits of ribosomal proteins - protein synthesis	●	●	●	●	●	○	○	○
		rpl2	ribosomal protein L2	large and small subunits of ribosomal proteins - protein synthesis	●	●	●	●	●	●	●	●
		rpl20	ribosomal protein L20	large and small subunits of ribosomal proteins - protein synthesis	●	●	●	●	●	●	●	●
		rpl23	ribosomal protein L23	large and small subunits of ribosomal proteins - protein synthesis	●	●	●	●	○	○	○	○
		rpl32	ribosomal protein L32	large and small subunits of ribosomal proteins - protein synthesis	●	●	●	○	●	●	○	○
		rpl36	ribosomal protein L36	large and small subunits of ribosomal proteins - protein synthesis	●	●	●	●	○	●	●	●
		rpl5	ribosomal protein L5	large and small subunits of ribosomal proteins - protein synthesis	●	●	●	●	●	●	●	●
	Small subunit	rps11	ribosomal protein S11	large and small subunits of ribosomal proteins - protein synthesis	●	●	●	●	●	●	●	●
		rps12	ribosomal protein S12	large and small subunits of ribosomal proteins - protein synthesis	●	●	●	●	●	●	●	●
		rps14	ribosomal protein S14	large and small subunits of ribosomal proteins - protein synthesis	●	●	●	●	●	●	●	●
		rps18	ribosomal protein S18	large and small subunits of ribosomal proteins - protein synthesis	●	●	●	●	○	○	○	○
		rps19	ribosomal protein S19	large and small subunits of ribosomal proteins - protein synthesis	●	●	●	●	●	●	●	●
		rps2	ribosomal protein S2	large and small subunits of ribosomal proteins - protein synthesis	●	●	●	●	○	○	○	○
		rps3	ribosomal protein S3	large and small subunits of ribosomal proteins - protein synthesis	●	●	●	●	●	●	●	●
		rps4	ribosomal protein S4	large and small subunits of ribosomal proteins - protein synthesis	●	●	●	●	●	●	●	●
		rps7	ribosomal protein S7	large and small subunits of ribosomal proteins - protein synthesis	●	●	●	●	●	●	●	●
		rps8	30S ribosomal protein S8, chloroplastic	large and small subunits of ribosomal proteins - protein synthesis	●	●	●	●	●	●	●	●

		rps9	30S ribosomal protein S9, chloroplastic	large and small subunits of ribosomal proteins - protein synthesis	●	●	●	●	○	○	○	○
Transcription (plastid-encoded RNA polymerase)		rpoA	alpha subunit of RNA polymerase	plastid-encoded RNA polymerase	●	●	●	●	●	●	○	○
		rpoB	beta subunit of RNA polymerase	plastid-encoded RNA polymerase	●	●	●	●	●	●	○	○
		rpoC1	beta' subunit of RNA polymerase	plastid-encoded RNA polymerase	●	●	●	●	●	●	○	○
		rpoC2	beta' subunit of RNA polymerase	plastid-encoded RNA polymerase	●	●	●	●	●	●	○	○
Translation		tufA	translational elongation factor Tu	GTP-dependent binding of aminoacyl-tRNA to the A-site of ribosomes during protein biosynthesis	●	●	●	●	●	●	○	○
		infA	translation initiation factor 1	Component of the 30S ribosomal translation pre-initiation complex	●	●	●	●	○	○	○	○

[a] *P. stagnora* plastid also includes 3 ORFs of unknown function

Supplementary Table 3. Annotation statistics for nuclear DNA assemblies of *P. zopfii* and related *Chlorellales*.

	***P. zopfii* gen. 1**	***P. zopfii* gen. 2**	***P. stagnora***	***P. cutis***	***Helicosporidium* sp.**	***C. variabilis***	***A. protothecoides***
**assembly length**	26,448,891	24,744,895	16,896,228	19,969,220	12,373,820	42,214,557	21,856,191
**genes**	6,884	6,381	7,041	6,884	6,033	9,780	7,014
**coding density (genes/Kb)**	0.26	0.26	0.41	0.34	0.49	0.23	0.32
**exons**	12,590	11,957	28,164	37,174	13,881	71,474	39,939
**Av exon/gene**	1.83	1.87	4.00	5.40	2.30	7.30	5.67
**Av exon size**	1,077	1,250	468	277	366	170	194
**Av intron size**	307	312	290	204	168	209	246

Supplementary Table 4. List of *P. zopfii* genes including transit peptide sequences as predicted by four localization prediction tools, for both plastid- and mitochondria-directed peptides. For each gene, the tool supporting the prediction and the related score (between parentheses) is reported.

		Num Targets	Gene ID	Pprowler	PredAlgo	TargetP	predSL	Num Predictors
P. zopfii 1	Plastid	2	g5108	• (0.90)	• (0.45)	• (0.88)	• (0.99)	4
			g3914		• (0.62)			1
	Mitochondrion	6	g1468	• (0.99)		• (0.83)	• (0.99)	3
			g6215	• (0.97)		• (0.68)	• (0.99)	3
			g5108		• (2.24)			1
			g3914				• (0.90)	1
			g3966				• (0.84)	1
			g4283				• (0.92)	1
P. zopfii 2	Plastid	2	g2780	• (0.91)	• (0.94)	• (0.41)	• (0.99)	4
			g4216		• (0.93)	• (0.38)		2
	Mitochondrion	8	g620	• (0.99)		• (0.73)	• (0.99)	3
			g2502	• (0.97)		• (0.68)	• (0.99)	3
			g4216	• (0.79)	• (0.56)			2
			g648			• (0.39)		1
			g2780			• (0.44)		1
			g3268			• (0.56)	• (0.99)	2
			g1664				• (0.97)	1
			g4295				• (0.99)	1

Supplementary Data

Supplementary Data 1. FASTA-formatted file of amino acid sequences of candidate NEPs for *P. zopfii* genotype 1 as resulting from the nuclear DNA assembly and annotation.

```
>g805.t1 [db_xref="UniProtKB/Swiss-Prot:Q10G20"] [product="DNA-directed RNA polymerase"] [note="similar to Oryza sativa subsp. japonica LOC_Os03g44484 gene"]
VLAAGAVTMFGDEEDVAIADDQDIDELDAWAVISSYFEERGLVRQQLDSFNDFINTGLQEIVDENNSII
ITPRNQHNGAQIEDEDRVYEIRFGQIYLSKPTFVEADGETAVLFPKEARLRNLTYAAPLYVDVEWRCGK
AAVEGGLDGGSSSAAPDADADVQTYEKVFLGDVPIMLRSDYCNLAGRSEADLADLGECPYDQGGYFVIN
GSEKVLIAQERMANNRVYVFKKAPPSRYSFASEIRSVAEGSTRLTSTMQCRLVGRAGNGGVVRVTLPYV
KADVPLLVVFRALGFVADRDVLEHVAYDLEDSEMLEALRASIEEALPIATKELALDYIGKRASVVGATR
DKRIRYAKDLLQKEFLPHVSVSPGAETRKAYFLGYAVHRLLLVALGRRPEDDRDHYSNKRLDLGGPLLA
NLFRQLFRKLARDARAVVQRAVDRGKDVNLTAAINKDTIGRGLKYSLATGNWGVLGGTQEMRAGVSQVL
NRLTFASTLSHLRRITSPIGREGKLAKPRQLHNSQWGMLCPAETPEGQACGLVKNLALMAYVSVGCAAA
PVLEFLEEWATEGLEEVSPAVVHKATKVFVNGAWVGVHRDPATLVRTLRSMRRQVDVNTEVGVVHDVRL
RELRLTTDHGRCCRPLFVVESGRLSIRKRDVASLGAGGAAGGWQRLVEEGHVEFVDVEEEETAMIAMQV
RDVADARKAAHDHLGRGADLIGGRHGLEGSSPPTSPSASSPTSTLAPRLPALATTYTHCEIHPAMILGV
CASIVPFPDHNQSPRNTYQSAMGKQAMGMYVTSYQVRMDTQGYVLYYPQKPLVTTRSMEYLRFRELPAG
INTIVAIMCYSGYNQEDSTMMNQSSIDRGIFRSIFLRSYRAEERRTAGGESERIERPDRDQTAGMRHGT
YDKLDDDGIAPPGTRVSGDDVVVGKTTPVGDGGANAAAGGGAARFARRDASTSLRHSESGVVDAVALTT
GADGQRFVKMRVRSVRIPQVGDKFASRHGQKGTIGITYSQEDMPFSRDGISPDLIINPHAIPSRMTIGH
LVEALMSKVAAVAGREGDATPFTSVTVDNISEALHREGHERRGWETLYHGHTGRRIIAPIFLNPTYYQR
LKHMVDDKIHARGRGPVQILTRQPVEGRARDGGLRFGEMERDCIISHGAAAFLRERLFEQSDAYRVHVC
ERCGLVAVANLKRNAFYCTGCKNSTRIAQVHMPYAAKLLFQELMAMCITPKLQFEIPKDPV
>g953.t1 [db_xref="UniProtKB/Swiss-Prot:Q010D7"] [product="DNA-directed RNA polymerase"] [note="similar to Ostreococcus tauri Ot10g01380 gene"]
MVEFETYSSAPVRKVKRIQFGLLDPDYIRRYSVAQIETSQTYENGRPKLGGLSDPRMGTMDRGVKCTTD
GMGVMECPGYFGHIELARPLYHALLTRTVLRVLRCVSFHNSKLMLLPDDPKRKGVCRIRNPERRLHAFA
AACAGKRVCEHTGGAQPAYRIEPGGLKITAEFAPPKGGDGEGADGAPPEGRAERRQELSADKALEILRR
ISDEDCKVLGFDVRYTRPDWMILTVLPVPPPPVRPSVMMDSSSRSEDDLTHQLSEILKANARLKRQEEA
GAPAHILAEFALLLQVHVTGYLDNTLPGVPRAKQRSGRPIKSISERLKGKHGRVRGNLMGKRVDFSART
VITGDPNLALDELGVPWSIALTLTFPEPVTPHNVERLRALVEEGPHPRPGRTGARYVVRDDGTRLDLRY
ARSERDRHLQPGYVVERHMIPGDVVVFNRQPSLHKMSMMGHRVRLLPHSTFRLNLSVTSPYNADFDGDE
MNMHMVQSHEARAEVREIMAVPANIVSPQANRPVMGIVQDSLLASRLLTSRDGFLERDELFNALLCLED
WDGRIPPPAVLKPRPLWTGKQLASMVLPRVSLERRAAWYRDGEPEGMSPTDSQVIIRDGVLVTGTLCKK
TLGASGGGLVHVTWMDHGPEAARAVLSQIQFVNNFWLLHHGFSIGIGDLIADAHTMGIINGIINRAKED
VKGLIARVQAGELEQQPGRTVMESFENQVNQVLNKARDDAGNRAQGSLQDSNNVVRMVTAGSKGSFINI
SQMIACVGQQNVEGKRIPFGFDARTLPHFTKDDYGPESRGFVENSYLRGLTPQEFFFHAMGGREGLIDT
AVKTASTGYIQRRLVKAMEDLQVRYDGTVRNGAGEVVQFLYGEDGMEGTAIEGQKIEPLNWDGAKMRRA
YAWDLDSPQLEASGILDTKTLERLRGDPHARAALDEELQAIEEDVRVLRRQVLTAGDASVNLPVNLKRL
LGAASRRFPAPDAPGGSGLGGAGALAAAPAAGAGTAKRKPHIFLGLCPTRVAARVAELLPSLRIVPGDD
PLSAEAQRNATLLFCAHVRFTLASKRVLCEHRLTPDAFEWLVGEIGARFAASAASPGEVVGTVAAQSIG
EPTTQMTLNTFHFAGVSAKNVTLGVPRLTEIINISKNIKTPSLTVFLLGKAARDKEAAKAVQCALEHTT
LRRVTAATEIHYDPDPRSTVVDKDREWVEAYWDLADPADADPARQSPWLLRVELARDMMVDKRLLLSEV
AERINADFGGELHCLFNDDNAEELVLRIRLLEDEQGGGDKGGGDGAGAEDDDDDVFLKRVEASMLSRVA
LQGIPGIRKVFLREARRTKLDDKGEFATGTEWVLDTEGVNLAAVLCHEDVDATRTTSNDIVEVLRVLGI
EAARGALLKELRGVIEFDGSYVNYRHLSALVDSMTRRGYFMAITRHGINRDETGPLHQASFEETVDILF
```

RAATYAERDDMSGVSENIIMGQTVPVGTGAFSLLVDEDRLKDAIELDYAFADDGSSAWGSGLTPGRTPG
RTPGTTPIRASPGSLAGMSPGPGGASPYVGDSDVAPVQPDFATVLADLAAILADFTAVFADLAAILADF
TAVQSDFTAVQSDLAAILADFTAVFADFAAVQPDLAAVLAGGQHQIALRERGHSQRTESERSQRRVHVR
TAPTLLARARARTSLAELARSVSTRCRRRSLIRAERHIVKPIVRLRFFTGIRGMLVPIDPPEGGEARAW
GAIELQGTIEPRATSSLRGLEGRLQLTIGNHNLDGSRVKVKKPLAVLCKETSASGMVSYKVVGVIRDRC
LFTARPRPIIEKAA
>g1468.t1 [db_xref="UniProtKB/Swiss-Prot:B2RFX8"] [product="DNA-directed RNA polymerase"] [note="similar to Selaginella moellendorffii rpoT gene"]
MARARAAVVPLRALLARALRRDAGGAAVVTGRRALDGGPLGIPANYSSRYASSSTPWRNDGVSTAPRPL
AAHASSPESGVGGVLVATLEPLGASASPHDSSSSAASNSADAVAREVAPSPGAPADGVRDLDAVSAREV
ADAAEASSSGADAAAAEVDSLSRAMRGVDVGVDAPLFSHRIAKAEAEGVTSGKPGAKPEAEAPKEARRE
ASARANAGEAAEAAGGSAKVTEGAQAAEAAEAAEGARAVEAAEGARSVEAAKAAKDASSSSASSSSSSA
LPSASASAPRPESQLSRLVGARRSGAEGRPAAHRRPKLRREASVLETMNWHSRFMDGSPEGEALRRSWR
RQVALETRAVELAAARYRRDAESAVSRGQGASLPVSRRLLLRWFEPLVAAIRHEQEQITARAPGLDRSV
YGPYLLLLAPEQLAVISMHTTLNMIMDADDRGTVGAGLKGMAGGAAEESNARAAGGADAGAGAGAGAGG
VALPGAGPDGAPVVCAPGGAAPLPLAGAGADLDAAALAAGSPPSFVPSPLPPPPSASGAPAPSFAGNAS
TPRPGGAVPGAVRMTRLSMAVGRAVESQVQLERLQALCSRVNRANRRVAKLRAEGAELRAALLRDGALP
EEGWARWREVGAELADAGEVMPHDPLSWFAPTEGVESRLSRLSVPAITRVGDVARGSQGTLAQIRKVTS
DDATLGDAWRLDVVAKVGAALIKLFLDTATVDVPKTRSGRGHALPALQSYHDAQRAAREREARETEQSN
ARASAGATGTLDGPMEASEAAADADAQGARIGAAVSSPPGLGAVSFAELPSVSLSFDAPGASRFPPPPA
CAPRSLELTGLPNERLTAASGGALELLGTAAQAASPSTSLVPSSSSSSSSSSSSFAGTSVEPAPYLSG
GTLLSPDESLSLQGSSPLPPPPSPPPIAPPKKGAGKRGKRAAASGGGASGSAPPPPDPALAALLSIEAQ
DVSRGRSTERAFWHALELLPDPSRRSRWKKYGLVFAHEEVARRVRPGEMAEAFVPVYTPMVIPPVPWQR
ADVGGHLTLRCHVMRTRGSHLQMRRLKAADAEREEGRGAGLSRVYAALNALGSTPWAIHHGVLSVVEAL
WEGGGGAAGLPARADFPIPPPVRAGFALRRVGESLALFDPGRDAERRSRAARKRLRRKNAELHSLRCDS
EHKLAVAREFRDEPAFYYPHNVDFRGRAYPMHPHLNHLGADLCRGMLTFAEAKPLGPKGMDWLCVQAAN
LWGGGVDKLPHAERVAWARENVERLRRNARDPLLLGDDWLDEMTPQARATFALGEAQARAARAARAAGG
GGAAGGGGGFVGGLAEAGGHLDEALSDPFAAGGVIAHVPVLGADGDAGGPSGPAAGASAAGSPSPSSPP
PPESFPPPSPVSPSATEAVRRAARLARALPGHALTWTEADAPFQFLAVCMEMDKALASGDPASYLSSLP
VHMDGSCNGLQHYAALGRDDDGGRAVNLVDAGRPQDVYTRIAERVRARVQKDAEAGVAHAQALLEATTV
DRKLVKQTVMTSVYGVTFVGARAQIGSRLRERGLADTPALYKVSCYAASITLGALHEMFSGAKDIMRWL
AECARAVARSGDTVGWTTPLGLPVVQPYRRLEKHHVRTLLQRLIIVDNNDNLPVMKQRQRTAFPPNFIH
SIDSTHMMMTATKCAKEGLSFAGVHDSYWTHAGDIDRMNQVLREQFVDLHSQPLLENLLEELQEAHPDV
EFPPVPTRGNLDLNEVKKSTYFFS
>g1582.t1 [db_xref="UniProtKB/Swiss-Prot:Q2T9S3"] [product="DNA-directed RNA polymerase III subunit RPC6"] [note="similar to Bos taurus POLR3F gene"]
MATDRNLETEILKLCQGDGISGTDLEARLPRVSAEERVKAINALLSADRLVPLTDSAGGVHFRASTSAD
RQRSSRLKGLTTDERFVYQLVEQASTMGIWTRDIKRRSNLPQNRINKILKTLDERRLIKSVKAVSNASR
KMYMLYDLEPAREVTGGPWYEAERFDGTFADAVGQAACKFIRERGSASVLEVSEFIKHEQITTEELTLE
DISNLLGALEYDGHLEWTPDVDARKRPKATGGRYTLAPSRIPVSTALSGAPCGVCPVIGECFEGGKVSP
SSCAYFQAWLEDY
>g2840.t1 [db_xref="UniProtKB/Swiss-Prot:A8I793"] [product="DNA-directed RNA polymerase"] [note="similar to Chlamydomonas reinhardtii CHLREDRAFT_113199 gene"]
PPSGRSIRVVASGEHEDAQILDADGLPHVGGVVWPGQHYYSCVDDLSGKVKFGKLKGEETAHVDQVAVI
GGRDKEITKVNIRMRYNRNPVIGDKFSSRHGQKGVLSRLYDDVDMPYAETTGIRPDLIINPHAFPSRMT
IGMLIESLTGKAGAVTGQFVDSTPFQSSEGGPKIPHEALGSALEAAGFTKNGGETLISGITGEPFDVDI

YMGVVYYQRLRHMVSDKFQVRSTGPINPLTKQPIKGRKFGGGIRFGEMERDSLLAHGAAYLLHDRLHSC
SDYSVSDVCRNCGLMISTMSLPLAGGAAGAVRAMDGAATQRNPYCRVCGTGKYVERVAVPYVFRYLVTE
LASMNIRCSLDVS
>g2879.t1 [db_xref="UniProtKB/Swiss-Prot:D0P2B6"] [product="DNA-directed RNA polymerase III subunit RPC8, putative"] [note="similar to Phytophthora infestans T30-4 PITG_20629 gene"]
MFTLATIEDHVLIAPQDLGLPTVACVEAALERAFLDKVIPEVGLVITLYDIVSIGPGLVHPSEGGAHRD
VRFRAVVFRPQIGEMLRGSVVRCEAARGVQVSLGFFDDIWIPSRCLPKGTTWDAKSGSFKWVPRPEEGE
EGEEAYDDADEDVEGAESFYIERGFDVRCKVVNLGFERRIAVQDAPGLQGGGLLGKQAGVPPPSVGRNG
PAGAASGPAGAAGGMPVGVTAAGGPAAGGDAAEEEDAEASAAVMVVTGVADGSGLGMVHWFPDDNYEEE
EEEPMET
>g3537.t1 [db_xref="UniProtKB/Swiss-Prot:A8IJY1"] [product="DNA-directed RNA polymerase subunit"] [note="similar to Chlamydomonas reinhardtii RPB9 gene"]
MSAPKLRFCPESNDLLYPRADRQRKVLTFVCRACGYVEDAPPSEWCVYRNEVHHSTREKLVVLQDVRSD
PTLPRTRDVRCPACGHDEAVFFSSSTEEGMTLFFNCAQCGHRWRDYV
>g3914.t1 [db_xref="UniProtKB/Swiss-Prot:A8J568"] [product="DNA-directed RNA polymerase (Fragment)"] [note="similar to Chlamydomonas reinhardtii RPA1 gene"]
MASSSVRKMSAKRVESPLVMDNDDLPVKDGLYDPAMGPLDGREICPTCGLGASCPGHMGHIELAVPVYN
PLVFGTLYRLLRSCCLHCFKLRMQAKAVEKIALRLERLSRGEDPDTDEVEEESGAEAGAGVAAEGAKGS
VAKPKRSSSSSSFPSDGDSSDGSTGSGSSLDAPGTPPRAPASPLSRPSSRVARGSARARVPTAQALEAM
QETISELFRKQPAGRCANCGAFSPTVKREGLSKLFLAPLPPKRAAANAARNVEVLPTLTRLRRPDDEVS
AADAYAVADGLLGDAARGAPGGASPPPPSSLSPSAASLPASLVLTATSVPGYAARLRAAARAAELDEPS
ALAVQASLEALEAARAVPPPAAAAIRAELDAALASIRGGFEVDASRKGAKAAARIAAGFARNDDDEEEE
EEEEKDKGGEGKAGASRQSAGEAMDVDASGAAWSSAPKPPSSASVPPSAPSSRAASAARTLPQYLTPAE
AWEIMRSVWAANERVLNLVYPTEAATRAWRRAKARAAASGRGDASSAFPRPSPLEAARASRAARRRGFE
ELFLRTIAVAPNRFRAPSRMGDRVYEHPQNTLLVRAINDTLELVRLRDLDPTREPATDARVLARWLSLQ
NTVNAFIDSSASDGRVDTPGIRQTLEKKEGLFRKNMMGKRVNFAARSVISPDPGLNGCEIGIPPYIAAR
LCFPERVTRFNLEKLREAVLAGPGGNPGAAAVEDRRGRVVSLDKLDRAGRERVARLLGVGLDHEEGRAH
AETAEEAERRGTPQGAAAAAAADGAPKGHVRFGGDDDDDEADGKVPGPGDADAAAAAAASASGALLSSS
VFPASSLSARPPPHVRSPEDPATSRFGGSMIVYRALEDGDVMLTNRQPTLHKPGVLAHRARIMRGERTI
RMHYANCSTFNADFDGDEINVHLPQDQLGRAEGYGIVSADRQFFAPTDGKPLRGLIQDHVVGATLLTMR
DRFLEPSLAAALVHEAVGVDCPGGWDVPSPEPAVYKPRVLCTGKQVLSAVLVHYTRDQLPFSCETGAKV
PAGEWGASSGEGRLVVRQSHLVAGVVDKAAFGGKGLLHLFHELYGARRTSLLTAALSRLFSAFLQRHGF
TCGLADVLLDEPAEERRTDALVTADGRAKGAAQEIVQVDLEHLLGKQKRQDDEERDEDDAKTPSGEKTG
KTKNKNKKKKSSPQDPDASEPSASSSSSSVAPALVRSALRARLRADPSLEALLDMKSSGSLHPLSSEVV
KACLPRGQVRPFRHNMMALMTVTGAKGSVVNFSQISCLLGQQELEGRRVPRMASGKTLPCFAPYDVGAR
SGGFVGDRFLSGLRPQEYYFHCMAGREGLVDTTVKTSRSGYLQRCLIKNLEALKVGYDGTVRDDADGSV
VQFAYGEDGIDPTYVGCLRAMPFFYHNMPQLHRHLTDFMGQDGKKKKEEGGEGDAEESVDVGTAAANGH
DAIVKTEGPVKMEHDDASEAKEKANAHRSGFSASSASLAVPLAPSSALLSATAAARARFLELARAAAEG
KLGEALFAGPGALRPLEGAPAARGAASEGYLAALAAGLAGGTLRAPAGGAKGADRELETLAREIDETHR
ALKKLGLEGEETEAKAKAKKKKNKGKDATAKRGDQNASSDASLVPSSSSLTAAFPRISLAPPTPLDPAA
FADLMTERFQRSMVPPGEAVGVIAAQSIGEPSTQMTLNTFHMAGRGEANVTLGIPRLREILMTAARELK
TPVMTLPALAADLVRRESPAALPARGHASPAQALAARLRRVRLAEALAGIRVQHVTLVQGVAAVEAAGS
KAKAKARAGEEGAEVSSSSLSRLYEVTLRFHPEEAYPAELGLTFGELARVTRVGFVPKLQAAVKAEIRR
ASTGSAVASVALAGLGGEEGAGLGTHGAGGANGGRALGPGASSLDGVDLADASSGRLSGRTVPPSASPD
DDDAAASDADDDEAEVNAENLDAKMERGGGAAEMATYESEDDEDRNLQDAAIRETGEEEEEDMQGIEDD
GKEVEGAFRGALDGSEGTAAAKNADKRASAAAPAGAAKAGALSSPAASRAASAKQASSSSSSSASSAGV

RVDRRARSVSVSLLVPYDAPALLMREVAERVAADVVVRGVKGIGKCYVLDGSGPGRDGTDAVQTDGMNV
PGAWRHADLCDVDRLGLNSPADFLRYYGVEAARASIMGEVGSVFGAYGIGVDPRHLSLIADAMTRGGGY
APLNRLGIQSSPAPFLKISFETAAAFLVAATLHGAVDSLQAPASRIVVGRPVGLGTGAVQVLQQEA
>g3966.t1 [db_xref="UniProtKB/Swiss-Prot:A8HSJ0"] [product="DNA-directed RNA polymerases I/II/III subunit"] [note="similar to Chlamydomonas reinhardtii RPB10 gene"]
MIIPVRCFTCGKVIGNKWETYLDLLQADYSEGCVVRAARGRGLSLVGDRATSAAEVGARPPRGTPLPRA
DRRSFLPPRSDAMDALGLNRYCCRRMVMTHVDLIEKLLNYNTLERAPQKPGH
>g4015.t1 [db_xref="UniProtKB/Swiss-Prot:C1FIF0"] [product="DNA-directed RNA polymerase"] [note="similar to Micromonas sp. RCC299 MICPUN_86086 gene"]
MVSAGVSPAGGVENVSSKASFKVGGVPSSLEQLQEELLGMDPAKLAEPIKEVTQKHELVPAFLKVRGLV
RQHIDSFDDFVDRELRKIVRAKGNAVVTCDASPDWFFRFLDIHVGEPQLEDTNRRCTPAECRLRDLTYS
APIYAEVEYVRGSEVVGRYRADRGSRGSGRAGGGSFPSGIGGGSVGIGGGTGQGPIYLGRLPIMLRSRR
CVLRRLDEAGLARAGECPLDPGGYFVVKGTEKVILIQEQLSKNRIVVDPDGRDGPVAAVTSSTHERKSK
THVGLKHGRVVLRHNAFSEDINLAVALRAMGATSDQEILALLGPEAALATLLAPTLQAARREADGTLCT
QHAALEWLGTKVRSSRPGGFGGGGGGGGGGAGGAGGGGGGAGAGSSRSGSASGAGSSSAGPRLTATGR
PMRTKADEARDVLANVVVCHVPAPGYDFDAKRRYLGLMARRLLAASVDPSAADDRDYYGNKRLELAGGL
LALLFEDLFKRTCADLKRAAELALSRAPGRSSGAFDAAKHVRTDALTLGLESALSSGNWTIRRFRMERR
GVTQVLSRLSFIAALGMMTRISSQFEKTRKVSGPRALQPSQWGMLCPADTPEGEACGLVKNLALLAHVT
TDADEAPVARAAAALGVVPASALAADEPGRGRSALVLLNGRVLGAHADPRLLPRALRSLRRRGRLASFV
SVWRGADGCVHVACDGGRVCRPLVVVDGTGRPRVGLAEVQAVRDGRETFEGLVRAGKVEFLDVNEENDS
LVAVREPQIVPGKTTHLEIAPFTLLGVVAGLVPYPHHNQSPRNTYQCAMGKQALGAIGFNQMTRADTLL
YLLDYTQRPLLSTRTIQLVGYDRLGAGQNAIVAVMSFSGYDIEDALVANRASLDRGFGRCALVRKHATS
LKRYPNRATDRIAAPPPPPPERPGRRRGPGGAPPARALSGGSGRFAALDADGIAEPGRPLGTGEVLVNK
QVPTVTRDILPAGGGGMGAGGGPGAPGGALLGGGMGVGGASLLSPPVPAAAAFRPAPVTWKGYAGERAT
VERVTLSSSDETPCAIKVMVRHARRPELGDKFSSRHGQKGVVGNIVQQMDMPFSERGLNPDLIMNPHGF
PSRMTVGKMIELIGSKAGALDGRTRLGSAFGELDGLAEEVSAIGETLVEHGFSYGGKDLLHSGITGEPL
QAYIFMGPVYYQKLKHMVIDKMHARARGPRVVLTRQPTEGRSHDGGLRLGEMERDCLIGYGASLLLLER
LMISSDQFEVSICTTCGLMGYHSAKTGHATCPVHGVTDAMATMKLPYAAKLLFQELQAMNIVPRLTLGE
A
>g4222.t1 [db_xref="UniProtKB/Swiss-Prot:A8J805"] [product="DNA-directed RNA polymerase II, 23 kDa polypeptide"] [note="similar to Chlamydomonas reinhardtii RPB5 gene"]
MDNVTRFYRIRKTCMELLRDRNYLVPQDELDLTKDEFRARFGDEPRKDDLTILVAREDPSDQLFVFFPE
ETKVGVKTMKVLAERMRNESVQRAIMVVEQSMTPFARQCLQEMQPRYLLEVFREEELLVNITKHVLVPE
HRVLSAEEKKTLLERYKVRDTQLPRIQLADPVARYYGVQRGQVVRIVRPSETAGRYVTYRLCV
>g4283.t1 [db_xref="UniProtKB/Swiss-Prot:Q01DV6"] [product="DNA-directed RNA polymerase"] [note="similar to Ostreococcus tauri Ot02g06220 gene"]
KRITFGVMSPSEMAAVSELHVFDRALYRMPGRLPASGGVLDPRLGVSNKRGICETCGHGLAACTGHWGY
VRLALPVFHVGYFKATLQTLQCICKRCSRVLVAESERRALLRGLRAQRAGERPQREAAFRRILDRCKRT
RVCPHCGADNGVVKKASGALKILHDPGPRAARLAAARSGGGGGGGGTATARTGAGAGAGGRSSKLAKAN
GGSIDLGRRGRGVGGNGDDDDDDDDDEGSDADAASESVSEASASSCSSSEDDEGGRAHRGRKGAGGGGK
AAAHASISAAARQEARNLPPRPPFAAREAPGSAAASLAGGAAADDLGEPLRRNEALRGCLVRAVDDLTP
LRVLWLLRAIPDEDLELLDLAARPEDLLACAVPVPPVSIRPSVEVDGGGGSNEDDVTVKLMQIVEVNGA
VREALARGLPAGALAEAWDFLQIQVAGLVNSDLPGVTGPMAPQGRPMRGLVQRLKGKQGRFRGNLSGKR
VDFSGRTVISPDPNLSVDQVAVPRGMASVLTFPQRAHDGNLADLRRRILRGADRHPGAAFVLYPSGDRV
FLRYGDRRRVAAALRPGDVVERHVDNGDVVLFNRQPSLHRQSIMAHRAVVAGPWRTLRFNECACAPYNA

DFDGDEMNIHVPQTEEARAEAASLAGVARNLCTPKNGEILVAATQDFLTSAFLVTSRDAFFTRSQTCQL
VGYSSGGRCALELPPPAVLAPVELWTGKQIFSAMLRPASSVAVAVTLETREKKCARGRTHMDPNDGYVC
FRNGELVCGHLGKSTLGGGNKAGLFQVLAADYSPSVAAEAMARLSRLSARAIGDLGFSIGVDDVAPGAA
LEAAKRAAVARGYAACAALETRYRQGTLPPLPGCDADATLENRVTGELNAVREAAAGACMAGLDRHNSP
LIMSTCGSKGSPINIAQMVAC
>g4907.t1 [db_xref="UniProtKB/Swiss-Prot:C1MLF5"] [product="DNA-directed RNA polymerase"] [note="similar to Micromonas pusilla CCMP1545 MICPUCDRAFT_55696 gene"]
YFLPAGILLKCFLDVSDLELYDRIVANAGTDSGHRSFVAARAELVLKQAARFGLTTRKRCLEYLGDLFR
VALNVPERWTKLQVGEYLLREHLFIHLSDPVDKFNLAVQMLDKLYALANSQCCDDNADANSHHELLLPG
HLLLKFMREQLETSMEVLVQQVKRDLERSPEMVNFSDQQYIRRCTERIPEVGQRFEYLLNTGNMISKGY
MDLSQVSGFTIVAEKLNYH
>g5108.t1 [db_xref="UniProtKB/Swiss-Prot:A9RSV5"] [product="DNA-directed RNA polymerase"] [note="similar to Physcomitrella patens subsp. patens PHYPADRAFT_177840 gene"]
MASSVSSSREALGLPVSSRGSISSELLLEDSGATPRQLAEFASLCASRLDRKRVEPGATVGAVGAQSIG
EPGTQMTLKTFHFAGVASMN
>g5935.t1 [db_xref="UniProtKB/Swiss-Prot:C1BUA8"] [product="DNA-directed RNA polymerase II subunit RPB4"] [note="similar to Lepeophtheirus salmonis RPB4 gene"]
MKEVFSKPSTRVLTVSEARVILEDYVAACRVRDADYRPNPLLQKGLEYAQRFATNRNRGAIGKIREILA
GKGCSARELALLGSLAPQTAEEARVLIPSLTEERFSDEDLTEMLREVATHAEFE
>g6196.t1 [db_xref="UniProtKB/Swiss-Prot:C1FDM4"] [product="DNA-directed RNA polymerase"] [note="similar to Micromonas sp. RCC299 ACR2 gene"]
MAWFPRQPRFRLDDLVNIGPPVKDDGPSLADVRVFPRECREAKLTYKGPISATIAYQIEGSPVVQQVTR
RLGAIPLMVKSRLCYLRNLSRAELVEKKEEAHEFGGYFICNGNERIIRLLVQNRRHYIMAMRRSAYRKR
GPSFTDMATLIRCVRPDEHSLTNRCHYLSDGTAVFAITLRRA
>g6215.t1 [db_xref="UniProtKB/Swiss-Prot:C1FDM4"] [product="DNA-directed RNA polymerase"] [note="similar to Micromonas sp. RCC299 ACR2 gene"]
YVSHFRSVHRGAFFTEMRTTAVRKLLPESWGFMCPVHTPDGSPCGLLNHLTAMCRVICEEPEEADEIEN
GFRMVLAGLGMRPVTASSGEEDPASSLIVQLDGKVLGRMPLEIANAAISQLRAIKAAKLEEEETLTPDG
RTIKLCGEEHSIPSHTEIAYIPFEKGGPFPGIFLFTQAARMVRPVVQNASRAAELIGSLEQTTMNIRCP
DGGSGGSSHLKFSHSEFHTAAMLSLIASLTPYSDFNQSPRNMYQCQMAKQTMGTPMQSIAHRPDNKIYR
IQTPQTPITRTKRYDAYHMDEYPNGTNTIVAVLAYTGYDMEDAMILNKSCVERGFAHASVYKTETINLN
QDKANN
>g6320.t1 [db_xref="UniProtKB/Swiss-Prot:Q01DV6"] [product="DNA-directed RNA polymerase"] [note="similar to Ostreococcus tauri Ot02g06220 gene"]
MEEDGAERTIEAGFAALDAPKEQFMVPNVPRKIKRITFGVMSPSEMAAVSELHVFDRALYRMPGRLPAS
GGVLDPRLGVSNKRGICETCGHGLAACTGHWGYVRLALPVFHVGYFKATLQTLQCICKRCSRVLVAESE
RRALLRGLRAQRAGERPQREAAFRRILDRCKRTRVCPHCGADNGVVKKASGALKILHDPGPRAARLAAA
RSGGGGGGGGAATARTGAGAGAGGRSSKLAKANGGSIDLGRRGRGVGGNGDDDDDDDDDEGSDADAASE
SVSEASASSCSSSEDDEGGRAHRGRKGAGGGGKAAAHASISAAARQEARNLPPRPPFAAREAPGSAAAS
LAGGAAADDLGEPLRRNEALRGCLVRAVDDLTPLRVLWLLRAIPDEDLELLDLAARPEDLLACAVPVPP
VSIRPSVEVDGGGGSNEDDVTVKLMQIVEVNGAVREALARGLPAGALAEAWDFLQIQVAGLVNSDLPGV
TGPMAPQGRPMRGLVQRLKGKQGRFRGNLSGKRVDFSGRTVISPDPNLSVDQVAVPRGMASVLTFPQRA
HDGNLADLRRRILRGADRHPGAAFVLYPSGDRVFLRYGDRRRVAAALRPGDVVERHVDNGDVVLFNRQP

SLHRQSIMAHRAVVAGPWRTLRFNECACAPYNADFDGDEMNIHVPQTEEARAEAASLAGVARNLCTPKN
GEILVAATQDFLTSAFLVTSRDAFFTRSQTCQLVGYSSGGRCALELPPPAVLAPVELWTGKQIFSAMLR
PASSVAVAVTLETREKKCGRGRTHMDPNDGYVCFRNGELVCGHLGKSTLGGGNKAGLFQVLAADYSPSV
AAEAMARLSRLSARAIGDLGFSIGVDDVAPGAALEAAKRAAVARGYAACAALETRYRQGTLPPLPGCDA
DATLENRVTGELNAVREAAAGACMAGLDRHNSPLIMSTCGSKGSPINIAQMVAC
>g6421.t1 [db_xref="UniProtKB/Swiss-Prot:B1Y7H2"] [product="DNA-directed RNA polymerase subunit beta'"] [note="similar to Leptothrix cholodnii (strain ATCC 51168 / LMG 8142 / SP-6) rpoC gene"]
CGLKETVVLADKLLQSGFRLATRAGISIAIDDMLVPAQKHTLIERAESEAKEIEQQYVSGLVTAGERYN
KVVDIWGKAGDEIGKKMMDHLKVEKTVDRNGKTVDQESFNSIYMMADSGARGSAAQIRQLAGMRGLMAK
PDGSIIETPITANFREGLNVLQYFISTHGARKGLADTALKTANSGYLTRRLVDVTQDLVVTEDDCGTDN
GMAMRALVEGGEVIESLRDRVLGRVAAIDVVHPETQAPLLAAGNMLDEDVLDVLEQAGVDEIKVRTPLT
CGTRFGLCAKRYGRDLGRGGLVNVGEAVGVIAAQSIGEPGTQLTMRTFHIGGAASRAAVASSVEAKSDG
I
>g6453.t1 [db_xref="UniProtKB/Swiss-Prot:A0YZ94"] [product="DNA-directed RNA polymerase II, large subunit, putative"] [note="similar to Lyngbya sp. PCC 8106 L8106_17912 gene"]
MARVRVFALFAVLMLVCASGAQAASARSLLQDSPSPAAPSPESESPAPPPSPEAAPPSPEAAPPSPETS
APSPSPETSSPSPSPETASPSPETSSPSPSPETSSPSPSPETSSPSPETSSPSPSPETSSPSPETPSPE
SVPSQISNLVEATPEAENSTPAAATPAPEAESPPPEAATPAPETESPSPEAATPSPETESPSPEAATPS
PEAESPSPEAATPSPEAESPSPEAASPSPEAESPSPIPSPPPSPISVPPPAPVTVLAAASPAPVTQVTQ
IVQNVYVNQTIVNEITNVVVNNPTNVQAIAIAIGDSATATASASANGASASASATTGSGSSPSSGSSSP
APATAENVFSYATSSASAMATLIQYGYPGIAAEAYASAIISLFQNNQTTAGSSTTIAAVLGIYSAYQNG
YEGSAVFAANRFAYAISNYAGVGNYIAQIIATLLTIILTIFNLSATGESTVIVVPLEYLS
>g6676.t1 [db_xref="UniProtKB/Swiss-Prot:A8I4L4"] [product="DNA-directed RNA polymerase II, 16.5 kDa polypeptide"] [note="similar to Chlamydomonas reinhardtii RPB8 gene"]
SRIVSRSDLLALDLTLDVNVEIYPLSPGERLAITVARTLNLDGTPSPDHYDAGLSTAGRPLLSDRFEYV
VHGRVFKVKGAKGAGGRAEAFVSFGGLLAHIVGDPARVGELGLDDNVFLLAKKV

Supplementary Data 2. FASTA-formatted file of amino acid sequences of candidate NEPs for *P. zopfii* genotype 2 as resulting from the nuclear DNA assembly and annotation.

```
>g147.t1 [db_xref="UniProtKB/Swiss-Prot:C1BUA8"] [product="DNA-directed RNA polymerase II subunit RPB4"] [note="similar to Lepeophtheirus salmonis RPB4 gene"]
MSGAPEEDIFSKPSTRVLTVSEARVILEDYVAACRVRDAEYRPNPLLQKGLEYAQRFATNRNRGAIGKI
REILAGKGCSARELALLGSLAPQTAEEARVLIPSLTEERFSDEDLTEMLREVATHAEFE
>g356.t1 [db_xref="UniProtKB/Swiss-Prot:D0P2B6"] [product="DNA-directed RNA polymerase III subunit RPC8, putative"] [note="similar to Phytophthora infestans T30-4 PITG_20629 gene"]
MFVLVTLEDQVCIAPQDLGLPTVPCVESALQRAFLDKVIPDVGLITALYDIVSIGPGLVHPSEGGAHRD
VRFRVVAFRPSIGEMLRGSVVRCDEARGVQVSLGFFDDLWIPPRCLPKGTSWDADAGAFRWIPAAEDEE
GEGGGEAAEEDLEEFFFDQGFDVRCKVVNLRYERRIAMQDAPGLQGGGLLGRQAGQPPPAAGRNGPTAA
NGAAPEAADEAAEEKSDVAVMTVTAVADGSGLGMVHWYAEDDEEGEAAEQMDQET
>g620.t1 [db_xref="UniProtKB/Swiss-Prot:Q8L6F9"] [product="DNA-directed RNA polymerase"] [note="similar to Physcomitrella patens rpoT1 gene"]
MARARAAVVPLRALLARALRRDAGGAAVVTGRRALDGGPLGIRGNYLARHASSSTPWRSDGASAAPRPR
AAHASSPDSGVGGVLVATLEPFGANAAPHDSSASSAANSAANSADAAALDGAAAPAAAPQPADAGAVRD
LDAVSAREAGESASASAAEASAAEAEAAAHGDSVSRAMRGVDLGADVPLFSHRVAKAESGGARAEGAGA
EAEGDAIAAGLRRPADAGEAAADADAAAEAARPRRRGRQSKAEAEEGAAAEGAAAEGAAGEGAPGGAP
PRGSAPRPQSQLSRLVDAMKEGHPAPHHRPKLRREASVLETMNWHSRFMDGSPEGEALRRSWRRQVALE
TRAVELAAARYRRDAESAVSRGQGAALPVSRRLLLRWFEPLVEAVRHEQEQITARVPGMDRSVYGPYLL
LLSPEQLSVIAMHTTLNMVMDADDRGAVGTNLKGMAGGDDGGGARAAAGGAGGTHAGAPADAAGGSVAA
DGSFLSAAPASPSPASGPLSRPGGAVPGAVRMTRLSMAVGRAVESQVQLERLQALCSRVNRANRRVAKL
RAEGAELRAALLRDGSLTEAGWARWREVGAELADAGEVMPHDPLSWFAPTEGVESRLSRLSVPAITRVG
DVARGSQGTLAQIRKVTSDDASLGEAWRLDVVAKVGAALIKLFLDTATVDVSRSRAGSGHALPALQAYH
DAQRAARERREAEEAAKEAHGGLEGAAAAGAAGGAPPAAPPGLGAVSFVEVTEGAPGGANGAAGGALGI
ANGTGEGAATASAGGSSGPAPEGSSSLAAFPGERLEATSGGAGRLPSGAEDAQLVGSAAQASGPGPRRG
GSAVEGPLAAEGRPSASFSPSPSSSYLSGGTLLSPEESLSLQGGSPLPPPPTPAPISPPKKGKKGRRGA
GVGPEGGPAGPDPALRALLAIEARDAERGRSTERAFWHALELLPDPHRRSRWKKYGLVFAHEEVARRVR
PGEMAEAFVPVYTPMVIPPVPWQRADVGGHLTLRCNVMRTRGSHLQMRRLKAADAERDEGRGAGLSRVY
AALNALGSTPWSIHRGVLSVVEALWEGGGGVAGLPARADFPIPPPVRAGFALRRVGESLALFDPGREAE
RRSRAARKRLRRKNAELHSLRCDSEHKLAVAREFKDEPAFYYPHNVDFRGRAYPMHPHLNHLGADLCRG
MLTFAEAKPLGPKGLDWLCVQAANLWGGGVDKLPHAERVAWARDNVERLRRNARDPLLLGRDWLEELDP
QQRRAFELQELTQSRGGARGGAGGGSGAGAGAGALEAEPGAPDASAPPPPSFPPQSPMSPSATEAVRRA
ARVARALPGSALAWTEADAPFQFLAVCMEMDKALASGDPASYLSALPVHMDGSCNGLQHYAALGRDDDG
GRAVNLVDASRPQDVYTRIAERVRAKVERDAEAGVVHAKALLEATTVDRKLVKQTVMTSVYGVTFVGAR
AQIGSRLRERGLADTPALYKVSCYAASITLGALHEMFAGAKDIMRWLAECARAVAKTGDTVGWTTPLGL
PVVQPYRRLEKHHVRTLLQRLIIVDNNDNLPVMKQRQRTAFPPNFIHSIDSTHMMMTAAKCAKEGLSFA
GVHDSYWTHAGDIDRMNQVLREQFVALHSQPLLENLLEELQEAHPDVEFPPVPARGNLDLNLVKQSTYF
FS
>g632.t1 [db_xref="UniProtKB/Swiss-Prot:A9T5Q2"] [product="DNA-directed RNA polymerase"] [note="similar to Physcomitrella patens subsp. patens PHYPADRAFT_191908 gene"]
AHIESFDYFLGRGLVEVIERLAGVEVDHPVTGERCRFWIESVNIGPPVKDDGPSLADVRVFPRECREAK
LTYKGPISATIAYQIEGSPVVQQVTRRLGAIPLMVKSRLCYLRHLSRAELVQKKEEAHEFGGYFICNGN
ERIIRLLVQNRRHYVMAMRRSAYRKRGPSFTDMATLIRCVRPDEHSLTNRCHYLSDGTAVFAITLRRAE
```

YFLPAGILLKCFLDVSDLELYDRIVADAGTDAGHRSFVAARAELVLKQAARFGLTTRTKCLEYLGDLFR
VALNVPERWTKLQVGEYLLREHLFIHLSDPGDKFNLAVQMLDKLYSLADSQCCDDNADANSHHELLLPG
HLLLKFMREQLETSMEVLVQQVKRDLERSPEMVNFSDQQYIRRCTERIPEVGQRFEYLLNTGNMISKGY
MDLSQVSGFTIVAEKLNYH
>g648.t1 [db_xref="UniProtKB/Swiss-Prot:A0YZ94"] [product="DNA-directed RNA polymerase II, large subunit, putative"] [note="similar to Lyngbya sp. PCC 8106 L8106_17912 gene"]
MARSRRFALLAALLLASQQPVFGIRCVWDFSPRNVGLRAVAQTHGLAGAPRARRAFRRGVPSSNGGEAG
LLSVXNLKQISQSVAAVNSNLDLTNINVGGSVSGPVSSSASLTISGPVTVTTPSPSPSPSPSPVPVPVL
SPSPVPVPVLSPSPVPVPVLSPSPVPVPVLSPSPVPVPVLSPSPALVSPPPPPPSPIPVISPVLPSTPS
PETIASPNPEPIPIPTPEPTPVPSSELSPVPSPAAPSPPAPESVQVPSPESAVPTPEAVPSPETYPPTP
ETVPSPETYSPSPEAVPSPETVPSPETYSPSPEAVPSPETYSPSPEAVPSPETYTPSPEAVPSPETYSP
SPEAVPSPETYSPSPETYSPSLEAVPSPETYTPSPEAVPSPETYTPSPEAVPSPETYSPSPETYSPSPE
AVPSPETYTPSPEAVPSPETYSPSPETVPSPETYTPSPEAVPSPETYSPSPETYSPSPETVPSPETYSP
SPEAVPSPETYSPSPEAVPSPEIYSPSPETYSPSLEAVPSPETYTPSPEAVPSPETYSPSPETYSPSPE
AVPSPETYSPSPEAVPSPETYTPSPEAVPSPETYSPSPETYSPSPEAVPSPETYTPSPEAVPSPETYSP
SPEAVPSPETYSPSPETYSPSPEAVPSPETYSPSPETYSPSPEAVPSPETYSPSPEAVPSPETYSPSPE
TYSPSPETVPSPETYTPSPEAVPSPETYTPSPEAVPSPETYSPSPETVPSPETYSPSPEAVPSPETYTP
SPEAVPSPETYTPSPEAVPSPETYSPSPETYSPSPEAVPSPETYSPSPEAVPSPESYTPSPEAVPSPET
YSPSPETYTPSPEAVPSPETYSPSPETVPSPETYSPSPETYTPSPETVPSPETYSPSPEAVPSPETYSP
SPEAVPSPEIYSPSPETYTPSPEDVPPPETYSPSPEAVPSPEIYSPSPETVPSPEAVPSPETYSPSPEA
VPSPETYTPSPEIAVP
>g764.t1 [db_xref="UniProtKB/Swiss-Prot:A8J805"] [product="DNA-directed RNA polymerase II, 23 kDa polypeptide"] [note="similar to Chlamydomonas reinhardtii RPB5 gene"]
MDNVTRFYRIRKTCMELLRDRNYLVPQDELEQTKEEFRQRFGDEPQKDDLTILVAREDPSDQLFVFFPQ
ETKVGVKTMKVLAERMRNEGVQRAIMVVEQSMTPFARQCLQEMQPRYLLEVFREEELLVNITKHVLVPE
HRVLSAEEKKTLLERYKVRDTQLPRIQLADPVARYYGVQRGQVVRIVRPSETAGRYVTYRLCV
>g915.t1 [db_xref="UniProtKB/Swiss-Prot:D0NLD2"] [product="DNA-directed RNA polymerase I, II, and III subunit RPABC2, putative"] [note="similar to Phytophthora infestans T30-4 PITG_12877 gene"]
YAEEAVDQLEEDGAPWEEGPDPDAEVVPESEGGVAILERAEPGAVPPEQRITTRYLTKYERARVLGTRA
LQISMNAPVMVDVGTETDPLEIAYKELREKKIPFTIRRYLPDGSFEDWGLDELAIPER
>g1664.t1 [db_xref="UniProtKB/Swiss-Prot:Q01DV6"] [product="DNA-directed RNA polymerase"] [note="similar to Ostreococcus tauri Ot02g06220 gene"]
KRITFGVMSPAEMASVSELHVFDRALYRMPGRLPAPGGVLDPRLGTSSKRGICATCGHGLAACTGHWGY
VRLSLPVFHVGYFRATLQTLQCVCKTCSRVLVAPPERRQLLRGLRAQRAGERPQREAAFRRVLDRCKRA
RTCPHCGADNGVVKKAPGALKILHDPGPRAARMAAARAAKAAGGAAAGGAGAGGAGAGASGKASASASS
KAGRSVAVGSDSGSGSDSDSVESVSEPSDDDEAGAAGAPARASPAASSLRAPVSAAQRAALRALPPRPP
YAAAPPVPGSVSASLAGGSSRDDLGEPLRRNEALGACLVRAVDDITPLRALWILRAIPDEDLELLDLAA
RPEDLLACAVPVPPVSIRPSVEVDGGGGSNEDDVTVKLMQIVEVNGAVREALARGLPAGALAEAWDFLQ
VQVAGLVNSDLPGVAGPLAPQGRPMRGLVQRLKGKQGRFRGNLSGKRVDFSGRTVISPDPNLAVDQVAV
PRGMASVLTFPQRVYEGNLADLRRRVLRGADRHPGAAFVLYPSGDRVFLRYGDRRRVAAALRPGDVVER
HVDDGDVVLFNRQPSLHRQSIMAHRAVVAGPWRTLRFNECACAPYNADFDGDEMNVHVPQTEEARAEAA
SLAGVARNLCTPKNGEILVAATQDFLTSAFLVTSRDAFFTRAQTCQLVGYASGGRCPLALPPPAVLRPV
ELWTGKQVFSALLRPSPAVGVAVTLETREKKCGRGQTHMDPGDGYVCFRNGALVCGHLGKSTLGGGNKA
GLFQVLAADYSPSAAAEAMARLSRLSARAIGDLGFSIGVDDVAPGAALAREKRRAVARGYAACADIEAR
YRAGTLPPLPGCDADATLENRVTGELNAVREAAAGACMAGLDRHNSPLIMSTCGSKGSPINIAQMVACV

GQQSVSGRRAPDGFFRRTLPHFPRGDKSPEGKGFVASSFYDGLNPTEFFFHTMAGREGLVDTAVKTAET
GYMSRRLMKALEDLYAHYDGSVRNAEGALLQTAYGEDTMDPAAMEGAEGEPLALGRLLGAVKALFPGGR
NRALWERERSARAGGDATAEAEGGAESRAGGPRGPSAEAPPAQARTRIPAIEVGRPLGWGEEEEEKEKE
GQGGQGGGDNAAGSASDASGNDTNAPGASGDAPCASGNLAIASSVGQHTPGTPEAFADVLRAVQAALRA
SPDGDPCLSTALQAGGEGDAAVSPATARELVPLPGALAAAAEAALRAALLAPPLAPGKRPGEWLMTGGE
EEEEEEEGEGEEEGLEEGTPMELETEGGEAATAAAAAAAAAAATDNDAVATAAAAAADASPSGRAAAGA
AEEGAGVAPPAAAAGDGAPSAAAAKGCSSARSSLPRPLAPPPPPRPLSRSDRLAALARVRASSSAAFRE
HLRAFFASMVSSVSSARAALSLPPSRRGPLSSELLLEDCGATPRQLAEFASLCASRLDRKRVEPGATVG
AVGAQSIGEPGTQMTLKTFHFAGVASMNVTQGVPRLKEIINAAKRISTPLIEAALEVDGSEQTARIVRG
RLERSALGDVARSVAVEVAGAHACVRVRLDAAKAAALQLEVDARAARRALLLAPKLRLKPAHVRLDGDD
SILVFPPKDGDRLEQLRALEAALPHVAVCGIPTVSRAVVNGQEPKDAKLKEIEREKELAWRRRHRAKGG
KKQEGDPPREAAGGTAENPPPAPAKRYKLLAEGSDLRAVMATPGVAGLRCTTNHVMETWRVLGIEAARG
SIIAEIQATMGAHGMSVDPRHIQLLADTMTHRGEVLGITRFGIAKMKDSVLMLASFEKTTDHLFDAAIH
GRADDVVGVSESIIVGIPMPTGTGLFKIAHDPVLEGKRGAQRTTERHRDEPAASRPTKPQRRQAALDAA
IPRRPAPVLAALGAGP
>g1878.t1 [db_xref="UniProtKB/Swiss-Prot:A8IJY1"] [product="DNA-directed RNA polymerase subunit"] [note="similar to Chlamydomonas reinhardtii RPB9 gene"]
MSAPKLRFCPESNDLLYPRADRQRKVLTFVCRACGYVEDAPPSEWCVYRNEVHHSTREKLVVLQDVRSD
PTLPRTRDVRCPACGHDEAVFFSSSTEEGMTLFFNCAQCGHRWRDYV
>g2225.t1 [db_xref="UniProtKB/Swiss-Prot:Q38859"] [product="DNA-directed RNA polymerase II subunit RPB11"] [note="similar to Arabidopsis thaliana RPB13.6 gene"]
IAFERDTKVADAGTFTIQREDHTVGNAVRLQLLRDDHVIFSGYRIPHPLEHRMVVRVQTDGQKTPLVAM
QESLAHINLQVQSLHGQFQQEVQRVRPTDVQ
>g2502.t1 [db_xref="UniProtKB/Swiss-Prot:D0MW98"] [product="DNA-directed RNA polymerase"] [note="similar to Phytophthora infestans T30-4 PITG_02420 gene"]
YVSHFRSVHRGAFFTEMRTTAVRKLLPESWGFMCPVHTPDGSPCGLLNHLTAMCRVICEEPEEADEIEN
GFRMVLAGLGMRPISASSGDEDPASSLVVQLDGKVLGRMPLKIANAAVSQLRAIKAAKLAEEEALTPDG
RTIKLCGEEHSIPAHTEIAYIPFEKGGPFPGIFLFTQAARMVRPVVQNASRAAELIGSLEQTTMNIRCP
DGGSGGSANLKFSHSEFHTAAMLSVIASLTPYSDFNQSPRNMYQCQMAKQTMGTPMQSIAHRPDNKIYR
IQTPQTPITRTKRYDAYHMDEYPSGTNTIVAVLAYTGYDMEDAMILNKSCVERGFAHASVYKTETINLN
QDKANNSGLRFGVPKDSKARGPVPHPIGAFGDKFPQVVPSPADSEAVAAKRVVASGEHEDAQILDTDGL
PHVGGVVWPGQHYYSCVDDLSGKVKFGKLKGEETALVDQVAVVGGRDKEITKVNIRMRYNRNPVIGDKF
SSRHGQKGVLSRLYDDVDMPYAEATGIRPDLIINPHAFPSRMTIGMLIESLTGKAGAVTGQFVDSTPFQ
SSEGGPKVPHEALGSALEAAGFTRNGGETLISGITGEPFDVDIYMGVVYYQRLRHMVSDKFQVRSTGPI
NPLTKQPIKGRKFGGGIRFGEMERDSLLAHGAAYLLHDRLHSCSDYSVSDVCRHCGLMISTMSLPLAGG
AAGAVRAMDGAAAQRNPFCRVCGTGKYVERVAVPYVFRYLVTELASMNIRCSLDVS
>g2780.t1 [db_xref="UniProtKB/Swiss-Prot:Q01FH9"] [product="DNA-directed RNA polymerase"] [note="similar to Ostreococcus tauri Ot01g06000 gene"]
MVAATLSAPRPSGGRRGDEGSSAAPPSSGKAGCAPASVPSASELPAPSSLDQLREELLGMDPAKLAAPI
KEVTQKHELVPAFLKIRGLVRQHIDSFDDFVDRELRKIVRAKGNAVVTCDASPDWFFRFVDIHVGTPQL
EDTNRACTPAECRLRDLTYSAPIYAEVEYVRGSEVVGRYRSDRRPGAGLGGGGIGIGGGTGQGPIYLGR
LPIMLRSRRCVLRGLDEAGLARAGECPLDPGGYFVVKGTEKVILIQEQLSKNRIVVDPDGRDGPVAAVT
SSTHERKSKTHVCLKHGKVMLRHNAFSEDVNLAIALRAMGATSDQEILALLGPEAALATLLAPTLQAAR
KEADGSLCTQHAALEWLGTKVRSSRPGGGPAGAPPAGAPAGRGPGGGAPGGPRLTSTGRPMRTRADEAR
DVLANVVVCHVPAPAYDFDAKRRYLGLMARRLLAASVDPSAADDRDYYGNKRLELAGGLLALLFEDLFK

RTCADLKRAAELALSRAPGRASGAFDAAKHVRTDALTLGLESALSSGNWTIRRFRMERRGVTQVLSRLS
FIAALGMMTRISSQFEKTRKVSGPRALQPSQWGMLCPADTPEGEACGLVKNLALLAHVTTDADEAPVAR
AAAALGVVPASALAADEPGRGRSALVLLNGRVLGAHADPRLLPRALRSLRRRGRLGCFVSVWRGADGCV
HVACDGGRVCRPLVVVDGTGRPRVGRDEVRAVRDGRETFEGLVRSGKVEFLDVNEENDSLIAVREPQIV
PGRTTHLEIAPFTLLGVVAGLVPYPHHNQSPRNTYQCAMGKQALGAIGFNQMTRADTLLYLLDYTQRPL
LSTRTIQLVGYDRLGAGQNAIVAVMSFSGYDIEDALVANRASLDRGFGRCALVRKHATSLKRYPNRATD
RIAAPPPPPPERPGRRRGPGGAPAPRALSGGSGRFAALDVDGIAEPGRPLGMGEVLVNKQVPTVTRDSV
LGPGQAAAGAPGAPPAPGAFPLGAAPGASPGGLPPPPAFRPAPVTWKGYAGERATVERVTLSSSDETPC
AIKVMVRHSRRPELGDKFSSRHGQKGVVGNIVQQMDMPFSERGLNPDLIMNPHGFPSRMTVGKMIELIG
SKAGALDGRTRLGSAFGELDGLAEEVGAIGETLVQHGFSYGGKDLLFSGITGEPLQAYIFMGPVYYQKL
KHMVIDKMHARARGPRVVLTRQPTEGRSHDGGLRLGEMERDCLIGYGASLLLLERLMISSDQFEVSICT
TCGLMGYHSAKTGHATCPVHGVTDAMATMKLPYAAKLLFQELQAMNIIPRLTLGDA
>g3268.t1 [db_xref="UniProtKB/Swiss-Prot:D0NBY8"] [product="DNA-directed RNA polymerase III subunit RPC5-like protein"] [note="similar to Phytophthora infestans T30-4 PITG_09425 gene"]
TMLLQYPLVGVLARPASWPTSDHLKLKPKHGRVQLETPVADEAGEAVQDAAPSRLELEGRSRPPESDLA
IGLVSDGALYVVPVDSVAAMRPSFSHLEKHEEPVRGRAQQLTQMSVQITRRETEAQIEARRRSYAYLAS
EEAREPWAEMRYHESESEAASLLVERLKAVDRGSVPYPMSTERYLEALLPRFVDEVEEIDAPHELTSRA
AAAPGGRPPSPASETQHPFPSEHLTAFEAALVEALRSESVMGPERIRAALSTRAQELPWIRDVVRASGA
EIAKAARSSSRVEEIRDRFVLRSSGGDDPIREVIIGILRKKDSMRKSEVQRAALEQGIDMTEHAYSKII
KELCTSAGPTWSFKKSA
>g3982.t1 [db_xref="UniProtKB/Swiss-Prot:Q1WMR3"] [product="DNA-directed RNA polymerase"] [note="similar to Coprinellus disseminatus RPB2 gene"]
MFGDEEDVAIADDQDIDELDAWAVISSYFEERGLVRQQLDSFNDFINTGLQEIVDENNSIIITPRNQHN
GAQLEDEDRVRFGQIYLSKPTFVEADGETAVLFPKEARLRNLTYAAPLYVDVEWRCGRTAVDGADEAGA
FGPDAAADVQTYEKVFLGDVPIMLRSDYCNLAGRSEADLADLGECPYDQGGYFVINGSEKVLIAQERMA
NNRVYVFKKAPPSRYSFASEIRSVAEGSTRLTSTMQCRLVGKAGSGGVVRVTLPYVKADVPLLVVFRAL
GFVADRDVLEHVAYDLEDAEMLEALRASIEEALPIATRELALDYIGKRASVVGATRDKRIRYAKDLLQK
EFLPHVSVSPGAETRKAYFLGYAVHRLLLVALGRRPEDDRDHYSNKRLDLGGPLLANLFRQLFRKLARD
ARAVIQRAVDRGKDVNLTAAINKDTIGRGLKYSLATGNWGVLGGTQEMRAGVSQVLNRLTFASTLSHLR
RITSPIGREGKLAKPRQLHNSQWGMLCPAETPEGQACGLVKNLALMAYVSVGCAAAPVLEFLEEWATEG
LEEVSPAVVHKATKVFVNGAWVGVHRDPATLVRTLRSMRRQVDVNTEVGVVHDVRLRELRLTTDHGRCC
RPLFVVENGRLSIRKRDVASLGAGGAAGGWQRLVEEGHVEFVDVEEEETAMIAMQVRDVADARKAAYDH
LHGAAHGDAAAAARGGSSASPLADAAADGQSAGARASGPAPRLPSLATTYTHCEIHPAMILGVCASIVP
FPDHNQSPRNTYQSAMGKQAMGMYVTSYQVRMDTQGYVLYYPQKPLVTTRSMEYLRFRELPAGINTIVA
IMCYSGYNQEDSTMMNQSSIDRGIFRSLFLRSYRAEERRAPSGESERIEKPDRDQTAGMRHGTYDKLDD
DGIAPPGTRVSGDDVVVGKTTPVGDGGANAAAGGSAARFARRDASTSLRHSESGVVDAVALTTGADGQR
FVKMRVRSVRIPQVGDKFASRHGQKGTIGITYSQEDMPFSRDGISPDLIINPHAIPSRMTIGHLVEALM
SKVAAVAGREGDATPFTSVTVDNISEALHREGHERRGWETLYHGHTGKRIVAPIFLNPTYYQRLKHMVD
DKIHARGRGPVQILTRQPVEGRARDGGLRFGEMERDCIISHGAAAFLRERLFEQSDAYRVHVCERCGLV
AVANLKRNAFYCTGCKNSTRIAQVHMPYAAKLLFQELMAMCITPKLQFEIPKDPE
>g4216.t1 [db_xref="UniProtKB/Swiss-Prot:A8HPP5"] [product="DNA-directed RNA polymerase I subunit"] [note="similar to Chlamydomonas reinhardtii RPA12 gene"]
MDAQRRSGLLFCPLTGSLLEVDPSRGVAASRASSFALPLSRVLGRERVVQDVDMADYARRYALEPLVRP
RAEADHAEQAIGARTRATVDEPCPKCGNPEMEFYTLQLRSADEGQTVFYECPKCGHKYSTNN
>g4295.t1 [db_xref="UniProtKB/Swiss-Prot:C1EA31"] [product="DNA-directed RNA polymerase"] [note="similar to Micromonas sp. RCC299

MICPUN_94662 gene"]
RRYSVAQIETSQTYENGRPKLGGLSDPRMGTMDRAIKCTTDGMGVMECPGYFGHIELARPLYHALLTRT
VLRVLRCVSYHNSKLMLLPDDPKRKAIARIRNPERRLHAFAAACAGKRVCEHTGGAQPAYRIEPGGLKI
TAEFAPPKGGDADGADGAPPEGRAERRQELPAERALEILRRISDEDCKVLGFDVRYTRPDWMILTVLPV
PPPPVRPSVMMDSSSRSEDDLTHQLSEILKANARLKRQEEAGAPAHILAEFALLLQVHVTGYLDNTLPG
VPRAKQRSGRPIKSISERLKGKHGRVRGNLMGKRVDFSARTVITGDPNLALDELGVPWSIALTLTFPEP
VTPHNVERLRALVEEGPHPRPGKTGARYVVRDDGTRLDLRYARSERDRHLQPGYVVERHMIPGDVVVFN
RQPSLHKMSMMGHRVRLLPHSTFRLNLSVTSPYNADFDGDEMNMHMVQSHEARAEVREIMAVPANIVSP
QANRPVMGIVQDSLLASRLLTSRDAFLERDELFNALLCLEDWDGKIPPPAVVKPRPLWTGKQLASMVLP
RVSLERRAAWYRDGEPEGMSPTDSQVIIRDGMLVTGTLCKKTLGASGGGLVHVTWMDHGPEAARAVLSQ
IQFVNNFWLLHHGFSIGIGDLIADAHTMGIINGIINRAKEDVKGLIARVQAGELEQQPGRTVMESFENQ
VNQVLNKARDDAGNRAQGSLQDSNNVVRMVTAGSKGSFINISQMIACVGQQNVEGKRIPFGFDARTLPH
FTKDDYGPESRGFVENSYLRGLTPQEFFFHAMGGREGLIDTAVKTASTGYIQRRLVKAMEDLQVRYDGT
VRNGAGEVVQFLYGEDGMEGTAIEGQKIEPLNWDGAKMRRAYAWDLDSPALEASGVLDAKTLERLRGDP
KARAALDEELQAIEEDVRTLRRQVLTSGDASVNLPVNLKRLLGAASRRFPAPDAGPGAAAAAPNGAAAG
GAPGKRTAHAYLGLCPTRVAARVTELLPSLRIVPGDDALSAEAQRNATLLFCAHVRFTLASKRVLCEHR
LTPDAFEWLVGEIGARFAASAASPGEVVGTVAAQSIGEPTTQMTLNTFHFAGVSAKNVTLGVPRLTEII
NISKNIKTPSLTVFLLGRAARDKEAAKAVQCALEHTTLRRVTAATEIHYDPDPRSTVVDKDREWVEAYW
DLADPADADPARQSPWLLRVELARDMMVDKRLLLSEVAERINADFGGELHCLFNDDNAEELVLRIRLLE
DEQSGGDKGGDGAGAEDDDDDVFLKRVEASMLSRVALQGIPGIRKVFLREARRTKLDDRGEFATGTEWV
LDTEGVNLAAVLCHEDVDATRTTSNDVVEVLRVLGIEAARGALLKELRGVIEFDGSYVNYRHLSALVDS
MTRRGYFMAITRHGINRDETGPLHQASFEETVDILFRAATYAERDDMSGVSENIIMGQTIPVGTGAFSL
LVDEGRLKDAIELDYAFADDGSSAWGSGLTPGRTPGRTPGTTPIRASPGSLAGMSPGPGGASPYVGDVG
FSPLSDGASFSPGYSPTSPAYSPTSPAYSPTSPAYSPTSPAYSPTSPAYSPTSPAYSPTSPAYSPTSPA
YSPTSPAYSPTSPAYSPTSPAYSPTSPAYSPTSPAYSPTSPAYSPTSPAYSPTSPAYSPTSPAYSPTSP
AYSPTSPAYSPTSPQYSPTSPQYSPTSPQYSPTSPQYSPTSPQYSPTSPQYSPTSPQYSPTSPQYSPTS
PQYSPTSPQYSPTSPQYSPEDNAK
>g4664.t1 [db_xref="UniProtKB/Swiss-Prot:A0YZ94"] [product="DNA-directed RNA polymerase II, large subunit, putative"] [note="similar to Lyngbya sp. PCC 8106 L8106_17912 gene"]
PEYVPSPEYVPSPEAVVPSPEYVPSPEGYTPSPEGTVPSPEYVPSPEGYTPSPEETVPSPEYVPSPEYV
PSPEAVVPSPEYVPSPEGYTPSPEGTVPSPEYAPSPEGYTPSPEETVPSPEYVPSPEYVPSPEYVPSPE
YVPSPEAVVPSPEYVPSPEAVVPSPEYVPSPEYVPSPEAVVPSPEYVPSPEAVVPSPEYAPSPEGYTPS
PEGTVPSPEYVPSPEGYTPSPEETVPSPEYVPSPESSSPSPEYVPSPEGYTPSPEGTVPSPEGYTPSPE
YVPSPEGYTPSPEGTVPSPEYVPSPEGYSPSPEETVPSPEYVPSPEGYSPSPEGYTPSTEYVPSPEWYT
PLPETPTAELTPSTESTPSPENVSVPSPVSLSSPCAGRSVIINETGTYAYDCDGSVVVIN
>g4837.t1 [db_xref="UniProtKB/Swiss-Prot:A8I4L4"] [product="DNA-directed RNA polymerase II, 16.5 kDa polypeptide"] [note="similar to Chlamydomonas reinhardtii RPB8 gene"]
MSRPIIFEDVFEITQIDPDGKKFDRVSRIVSRSDLLALDLTLDVNVEIYPLSPGERLAITVARTLNLDG
TPSPDHYDAGVSTAGRPLLSDRFEYVVHGRVFKLKGAKGAGGKAEAFISFGGLLAHVVGDPARERSPRS
RLRRSPAMQSALATLARRAAAARPAAASLHTGVEIVGSVAPTYQPWFDQAFELGKHDPRTRRGKVYKGS
FGKARPKSKPLHPWDPKTWEDPTALRIPPPPPPPSAPGATRPASQ
>g5239.t1 [db_xref="UniProtKB/Swiss-Prot:A8J568"] [product="DNA-directed RNA polymerase (Fragment)"] [note="similar to Chlamydomonas reinhardtii RPA1 gene"]
MQQSGLHSKEVTTAQVTAVRFGFFSDDEVRKMSAKRVESPLVLDNDDLPVKDGLYDPAMGPLDGREMCP
TCGLGASCPGHMGHIELAVPVYNPLVFGTLYRLLRSCCLHCFKLRMQDKEVQKVALRLERLARGDDPDA
ETDAGSDAAAAEERGDATAAGSAAAAGERGDATAPRKAGKKSSASGGSSSDGEASDGSTGSGSSLDAPG

TPPRAPAPPLSRPSAHARRTRAPTAQALEAMQETISELFRKQPAGRCANCGAFSPAVKREGLSKLFLAP
LPPKRAAANAARNVEVLPTLTRLRRPAEEVSAADAYATADGLLGNAKAGDAHATADGRLGDAKAADAAP
AALPPALVLTATSVPGYAARLRAAARAAELDEPQALAVQAALDALEASHAVPAAAAAAIRAELDGALAG
LRGGYVVDEPLKGAKAAARAAAGLDGAAAAGATSGLPDEDAAGMDVDADADADADAPAAGAAARAARDR
AAAASTLPQYLTPAEAWEIMRSVWAANERVLNLVYPTEAATRAWRRARARVARAGGSGRAGLAQAPSPL
EAARAARAARRRGFEELFLRTLAVAPNRFRAPSRMGDRVYEHPQNTLLVRAINDNLELVRLRDLDPSRE
PATDARVLARWLSLQNTVNAFIDSSASDGRVDTPGIRQTLEKKEGLFRKNMMGKRVNFAARSVISPDPG
LNGCEIGIPPYIAARLSFPERVTRFNLEKLREAVLAGPGGNPGAAAVEDRRGRVISLDKLDRAGRERVA
RLLGVGLENEEGRRDGEAYGAAAPGAQQRPGKAHVRFGDDDDEGEGGGAAAAAAAAAAAAPSPTPDDLE
AAFADAASLASRPPPHVRTPEDPATSRFGGSMIVYRALEDGDVMLTNRQPTLHKPGVLAHRARIMRGER
TIRMHYANCSTFNADFDGDEINVHLPQDQLGRAEGYGIVSADRQFFAPTDGKPLRGLIQDHVVGATLLT
MRDRFLEPALAAALVHEAVGVDCPGGWDVPAPEPAVYKPRVLCTGKQVLSAVLVHYTRDQLPFSCETGA
KVPAAEWGAHSGEGKLVIRQSHLVAGVVDKAAFGGKGLLHLFHELYGARRTSLLTAALSRLFSAFLQRH
GFTCGLADVLLDEPAEDRRAEALTTADGRAKGAAQEIVQVDLEHLGGARTDEDDRGEGKAGKSGSKGSA
SFPPALVRAALRSRLRADPSLEALLDMKSSGSLHPLSSEVVKACLPRGQVRPFRHNMMALMTVTGAKGS
VVNFSQISCLLGQQELEGRRVPRMASGKTLPCFAPYDVGARSGGFVGDRFLSGLRPQEYYFHCMAGREG
LVDTTVKTSRSGYLQRCLIKNLEALKVGYDGTVRDDADGSVVQFAYGEDGIDPTYVGCLRAMPFFYHNM
PQLHRHLTDCMGKQNAGGETAVVERSEKAGEGALSPTSAAATTAPIAPSGALLAATARARARFLALARA
AADGKLGADLAERLEPLEVAQAARGAASEGYLSALAAGLAGGTLRAPAQGRKGADHELATLVREVEETR
KTLQKLGVDVGERDGPKAGRKGGKGAEGASAASSVPVSSFPRVSLAPPAPLDPASFADLMTERFQRSLV
PPGEAVGVIAAQSIGEPSTQMTLNTFHMAGRGEANVTLGIPRLREILMTAARELKTPVMTLPALPAEAV
RRDSPGALPARGDATPALALAARLRRVRLAEALAGIRVEHVTLVQGVAAVEAKGGSKGKGEGGVGSVQT
GLSRLYEVTLRFHADDAYPAELGLTFAELARATRAGFVPKLQAAVKAEIRRASTGSAVASVALAGLGGE
EGAGAPAAPGARAARPADGGDLDDPAARRAAPAAQSPDDDDAAASDEDDDEAEANPENLDAKMERGGGA
AEMATYESEDDDDKHLQGEAIRETGEREEEDMEGLEDDAREVEGEIERGLAGEAARAASPAQASSSARK
AAAPAAAASARGSAASPPPPSSSSSSSGVRVDRRSRSVSVSLLVPHDAPALLMREVAERVAADVVVRGV
KGIGKCYVLDGSGPGRDGTDAVQTDGMNVPGAWRHADLCDVDRLGLNSPADFLKYYGVEAARASIMGEV
GSVFGAYGIGVDPRHLSLIADAMTRGGGYAPLNRLGIQSSPAPFLKISFETAAAFLVAATLHGAVDSLQ
APASRIVVGRPVGLGTGAVQVLQQEA
